# Marine forests of the Mediterranean-Atlantic *Cystoseira tamariscifolia* complex show a southern Iberian genetic hotspot and no reproductive isolation in parapatry

**DOI:** 10.1038/s41598-018-28811-1

**Published:** 2018-07-11

**Authors:** Ricardo Bermejo, Rosa M. Chefaoui, Aschwin H. Engelen, Roberto Buonomo, João Neiva, Joana Ferreira-Costa, Gareth A. Pearson, Núria Marbà, Carlos M. Duarte, Laura Airoldi, Ignacio Hernández, Michael D. Guiry, Ester A. Serrão

**Affiliations:** 1 Earth and Ocean Sciences Department, Ryan Institute and School of Natural Sciences, National University of, Ireland Galway, H91 TK33 Ireland; 2 Departamento de Biología. Facultad de Ciencias del Mar y Ambientales, Universidad de Cádiz, 11510 Puerto Real Cádiz, Spain; 3 Center of Marine Sciences (CCMAR), CIMAR Laboratorio Associado, Campus de Gambelas, Universidade do Algarve, 8005-139 Faro, Portugal; 4 Dipartimento di Scienze Biologiche, Geologiche e Ambientali. Università di Bologna, Piazza di Porta S. Donato 1, Bologna, Italy; 5 Department of Global Change Research, IMEDEA (CSIC-UIB), Institut Mediterrani d’Estudis Avançats, Miquel Marquès 21, 07190 Esporles Illes Balears, Spain; 6 King Abdullah University of Science and Technology (KAUST), Red Sea Research Center (RSRC), Thuwal, Saudi Arabia; 7 Instituto Universitario de Investigaciones Marines (INMAR), University of Cadiz, 11510 Puerto Real Cadiz, Spain; 8 AlgaeBase, Ryan Institute, National University of Ireland, Galway, H91 TK33 Ireland

## Abstract

Climate-driven range-shifts create evolutionary opportunities for allopatric divergence and subsequent contact, leading to genetic structuration and hybrid zones. We investigate how these processes influenced the evolution of a complex of three closely related *Cystoseira* spp., which are a key component of the Mediterranean-Atlantic seaweed forests that are undergoing population declines. The *C. tamariscifolia* complex, composed of *C. tamariscifolia s.s*., *C. amentacea* and *C. mediterranea*, have indistinct boundaries and natural hybridization is suspected. Our aims are to (1) infer the genetic structure and diversity of these species throughout their distribution ranges using microsatellite markers to identify ancient versus recent geographical populations, contact zones and reproductive barriers, and (2) hindcast past distributions using niche models to investigate the influence of past range shifts on genetic divergence at multiple spatial scales. Results supported a single, morphologically plastic species the genetic structure of which was incongruent with a priori species assignments. The low diversity and low singularity in northern European populations suggest recent colonization after the LGM. The southern Iberian genetic hotspot most likely results from the role of this area as a climatic refugium or a secondary contact zone between differentiated populations or both. We hypothesize that life-history traits (selfing, low dispersal) and prior colonization effects, rather than reproductive barriers, might explain the observed genetic discontinuities.

## Introduction

Climate changes during the Quaternary glaciations (c. 2.6 Myr to the present) caused periodic fluctuations in sea-level, sea ice and sea-surface temperature that have resulted in radical species range-shifts, that left genetic signatures in many European species^1^. Range expansions and contractions, and resulting patterns of refugial isolation, colonization and secondary contact have similarly structured the genetic and evolutionary traits of many marine species^2–4^. Populations isolated in climate refugia for long periods develop a genetic signature including more unique alleles^3,5^. On the contrary, outside stable areas, extinction/recolonization dynamics and associated bottlenecks typically result in eroded gene-pools, characterized by lower regional genetic diversity and originality^6^. High allelic richness can also arise by secondary contact between formerly vicariant, differentiated lineages^7^. Differentiating between refugia or admixture as causes of genetic hotspots is possible by analysing the global distribution of private alleles along ranges. Predictions that can be tested include: (i) secondary contact (admixture) zones with high allelic richness and low singularity; (ii) leading edges with lower diversity and differentiation; (iii) refugia with high and unique genetic diversity; and (iv) trailing edges with low diversity and high differentiation.

The effects of range shifts on creating ancient vicariant refugia and more recent admixture zones are particularly interesting to investigate in complexes of closely related species that are still partially reproductively compatible^8–10^. In such complexes, shallow genetic divergence, incomplete isolation and partially overlapping ranges and morphological traits can be particularly challenging to tackle. The very speciose brown algal genus *Cystoseira* is particularly interesting in this respect as it contains several complexes of closely related genetic entities with difficult taxonomic resolution^11^ that might hypothetically be influenced by hybrid zones with introgressive effects.

*Cystoseira* forests are among the most important foundation species in the Mediterranean and warm-temperate northeast Atlantic. They create complex habitats and are essential for biodiversity and ecosystem functioning^12–14^. Over the last decades, Mediterranean *Cystoseira* populations have suffered a general decline due to anthropogenic pressures^15,16^ that is likely to intensify under climatic change^17–19^. Therefore, most of these species have been protected (Annex II of the Barcelona Convention, COM/2009/0585 FIN; Annex I of the Bern Convention), and additional reforestation and protection measures have been recommended^20^. In the Mediterranean Sea, about two-thirds of ca. 28 species are considered to be endemic^11,21^, and new species are regularly added^22^. The genus is considered to be in the process of active speciation in the Mediterranean, but patterns and drivers of diversification remain poorly understood, partly due to morphological plasticity^23–25^ and shallow genetic divergences^11^. The recent development of high-resolution microsatellite markers^26,27^ is finally allowing the analysis of evolutionary processes at the species/population interface for some *Cystoseira* lineages. This may shed new lights on the role of climate-driven range-shifts and of putative introgressive processes in the evolution of this genus.

This study focuses on three closely related species, herein collectively referred to as the *C. tamariscifolia* complex: C. *amentacea* (C. Agardh) Bory (including the nominal variety *amentacea* and var. *stricta* Montagne), *C. mediterranea* Sauvageau, and *C. tamariscifolia* (Hudson) Papenfuss^25^. The complex belongs to the mtDNA clade V of Draisma *et al*.^11^, which also includes other ecologically important species such as *C. brachycarpa* J. Agardh, *C. crinita* Duby and *C. zosteroides* (Turner) C. Agardh. The three putative species differ in their general distributions, but overlap in some areas such as the Alboran Sea and Sicily^21,28–30^. *C. tamariscifolia* is distributed throughout the Atlantic from the British Isles to Mauritania (including the Macaronesia Islands), and in some Mediterranean areas under Atlantic influence (southern Spain, Sicily, Morocco, Algeria and Tunisia). The other two species are mainly restricted to the western Mediterranean, the Adriatic, and the Aegean Seas. Given the geological history of the Mediterranean Sea, Atlantic *C. tamariscifolia* has been hypothesized to be the ancestor of the other two species, which would have evolved independently from one another^25^. The extent to which these putative species are genetically distinct and reproductively isolated has never been investigated genetically, but natural ongoing hybridization has been suspected based on morphologically intermediate individuals^24,25,31^.

This study asks whether these species within the *C. tamariscifolia* complex form discrete genetic pools and how their divergence may have been shaped by past range shifts and hybridization at contact zones. To address this question, we analyse the genetic structure and diversity of this species complex throughout its distribution and integrate it with modelled past range shifts during climate extremes (the LGM) using SDMs. We use these results to identify genetic signatures of past range shifts and possible hybridization at contact zones between the three species. To further distinguish the alternative hypotheses of distinct species that admix when in contact versus a single species with morphotypes that vary among habitats, we analyse in detail a zone in the Iberian Peninsula containing parapatric populations of *C. amentacea* and *C. tamariscifolia*.

## Methods

### Ecological niche modelling

#### Sources of C. tamariscifolia, C. mediterranea and C. amentacea records

Georeferenced occurrences were compiled from our sampling surveys, plus 88 available publications and three databases: Algaebase^21^, Marine Forest (http://www.marineforests.com/) and the Global Biodiversity Information Facility (http://www.gbif.org/). A resolution of 5 arc minutes (~ 9.2 km) was used to georeference species data and climatic variables. We obtained 259 occurrence records for *C. amentacea* (which collapsed into 171 unique cells), 154 for *C. mediterranea* (80 cells), and 580 for *C. tamariscifolia* (315 cells) (see Supplementary S1.1 in Supporting information). Because the reproductive isolation between the three morphospecies is uncertain, the occurrences of these species were also pooled (993 occurrence records, 551 cells) to develop models including all entities, based on the hypothesis of a single “species complex” rather than three species. The coastline to 20 m depth was inferred from the 30 arc-seconds General Bathymetric Chart of the Oceans (http://www.gebco.net/). For the LGM coastline, a mean sea-level change of −116 m was considered.

#### Environmental variables

Maximum and minimum sea surface temperature (SST) under present conditions were obtained from the Bio-ORACLE dataset^32^ and tested with Pearson’s correlation coefficient (r < |0.7|). Variables for the LGM were obtained from the Coupled Model Intercomparison Project Phase 5 (CMIP5; http://cmip-pcmdi.llnl.gov/cmip5/). The uncertainty produced by the variability among the ocean general circulation models^33^ was reduced by using a multi-model ensemble with five models from the CMIP5 LGM experiment: CCSM4, CNRM-CM5, NASA-GISS-E2-R, IPSL-CM5A-LR, and MIROC-ESM.

#### Present and past SDMs

The predictions for the present and the LGM for the three *Cystoseira* species individually, and for the species complex as a whole, were obtained using an ensemble approach. “biomod2”^34^ was used to perform six presence-absence algorithms: generalized additive model, flexible discriminant analysis, generalized boosting model, generalized linear model, randomForest and multiple adaptive regression splines. Two sets of pseudo-absences were used, extracted at random with the same number of pseudo-absences as presence cells. We performed 10 iterations for each model and sets of pseudo-absences. In each iteration, data were split into a calibration (70%) and a validation set (30%). Thus, 120 models were computed for each species (2 pseudo-absence sets × 6 modelling techniques × 10 iterations). Model accuracy was assessed using the area under the receiver operating characteristic (ROC) curve (AUC), ROC-derived sensitivity (presences correctly predicted) and specificity (absences correctly predicted)^35^, and the true skill statistic (TSS^36^) considering a threshold that optimized ROC and TSS scores^34^. Two ensembles with the models that obtained TSS >0.7 through two different algorithms were computed: the mean of the probabilities and the average of the binary predictions. Finally, a consensus prediction averaging the two ensembles for each species was obtained. Ensembles were hindcasted to the LGM. The uncertainty in past projections was assessed computing a clamping mask, identifying the different values for each variable between the training range and the past climate. We also estimated the relative importance of each variable by the correlation between the full model and a model rearranged without one variable. After three iterations, the mean for each variable was calculated to obtain an importance value from 0 to 1 (the highest importance)^34^. Finally, the spatial overlap among the hindcasts for the LGM based on the three species was computed after reclassifying the probabilities into binary predictions according to the TSS-derived threshold of each ensemble. All analyses were performed in R^37^.

### Sample collection

Sampling aimed to cover most of the known distribution range of the three species studied, with a special focus on the Atlantic-Mediterranean transition zone, a contact zone between the mostly Atlantic *C. tamariscifolia* and the Mediterranean *C. amentacea* var. *stricta* (Table 1). Some coastlines could not be sampled due to travelling constraints (namely in Mediterranean Africa). To assess whether species were reproductively isolated in the absence of geographical (allopatric) hybridization barriers, a parapatric zone in Almeria, Southern Iberia, was identified between *C. amentacea* and *C. tamariscifolia* at “El Playazo” (Pi and Ps, Table 1), where the species co-occur but show depth range differences. At this site, individuals of *C. amentacea*, identified based on the presence of multiple cauloids (basal parts of the thallus that emerge from the holdfast), occurred close to the surface (Pi = parapatric intertidal) forming a dense meadow, while individuals of *C. tamariscifolia*, identified as those with a single cauloid, were scattered at depths between 1 and 2 m (Ps = parapatric subtidal) (see Supplementary S2).

**Table 1:** Summary of genetic diversity indices for *Cystoseira* spp. in all studied locations. Pop - Population Code; Name - Locality name; Latitude and Longitude in WGS84 coordinates; n - Number of genotyped individuals; A*15 - Mean allelic richness standardised to the smallest sample size (15) (±SE); Pa*15 - Mean number of privative alleles standardised to the smallest sample size (15) (±SE); He - expected heterozygosity (±SE); c*F*is - Inbreeding coefficient after correction for null alleles; Bold F_IS_ values are significant.

Pop	Name	Latitude	Longitude	n	A*_15_	Pa*_15_	He	c*F*_IS_
FN●	Finavarra	53.1582°N	9.1207°W	29	1.22 ± 0.16	0.00 ± 0.00	0.018 ± 0.012	−0.013
Po●	Polzeath	50.5858°N	4.8820°W	32	1.57 ± 0.44	0.00 ± 0.00	0.159 ± 0.101	0.020
Ro●	Roscoff	48.7294°N	4.0108°W	44	1.97 ± 0.29	0.21 ± 0.21	0.214 ± 0.093	0.136
Nj●	Noja	43.4962°N	3.5247°W	24	2.82 ± 0.37	0.14 ± 0.14	0.504 ± 0.070	**−0.161**
CV●	Cabo Vidio	43.5709°N	6.1755°W	32	3.03 ± 0.67	0.08 ± 0.08	0.378 ± 0.115	0.051
Pr●	Porcia	43.5676°N	6.8754°W	24	2.74 ± 0.66	0.00 ± 0.01	0.374 ± 0.120	0.006
VC●	Viana do Castelo	41.6993°N	8.8567°W	47	1.83 ± 0.21	0.00 ± 0.00	0.198 ± 0.073	**0.270**
Er●	Ericeira	39.1335° N	9.3882°W	24	6.00 ± 0.96	1.09 ± 0.81	0.609 ± 0.080	0.074
Od●	Odeceixe	37.4388°N	8.8040°W	15	4.83 ± 1.04	0.51 ± 0.35	0.587 ± 0.087	**0.221**
Sg●	Sagres	37.0082°N	8.9485°W	31	8.36 ± 1.45	0.74 ± 0.32	0.716 ± 0.074	−0.013
Ab●	Albufeira	37.0761°N	8.2769°W	48	5.73 ± 0.75	0.43 ± 0.31	0.705 ± 0.039	**0.058**
Ca●	Cádiz	36.4776°N	6.2644°W	31	6.53 ± 0.82	0.62 ± 0.33	0.667 ± 0.088	0.066
Ta●	Tarifa	36.0596°N	5.7197°W	32	9.80 ± 1.24	0.77 ± 0.36	0.789 ± 0.040	0.048
Ce●	Ceuta	35.9028°N	5.2962°W	31	9.45 ± 1.48	0.75 ± 0.16	0.842 ± 0.022	**0.135**
Cb●	Calaburras	36.5060°N	4.6397°W	32	11.47 ± 1.38	1.32 ± 0.25	0.849 ± 0.032	**0.085**
He●	Herradura	36.7361°N	3.7576°W	30	9.63 ± 1.48	1.00 ± 0.56	0.820 ± 0.030	**0.140**
GV★	Guardias Viejas	36.6949°N	2.8496°W	27	8.72 ± 0.56	0.72 ± 0.26	0.810 ± 0.012	**0.131**
Pi★	Playazo intertidal	36.8592°N	2.0032°W	31	8.82 ± 1.02	0.71 ± 0.39	0.784 ± 0.043	**0.122**
Ps●	Playazo subtidal	36.8592°N	2.0032°W	32	8.40 ± 0.90	0.69 ± 0.37	0.806 ± 0.029	**0.138**
Cq★	Calblanque	37.6055°N	0.7206°W	32	6.11 ± 1.17	0.69 ± 0.39	0.688 ± 0.069	0.026
SP★	Santa Pola	38.1964°N	0.5147°W	28	1.68 ± 0.38	0.00 ± 0.00	0.140 ± 0.066	0.068
PM■	Punta de la Mora	41.1262°N	1.3443°E	31	4.56 ± 1.05	0.64 ± 0.59	0.591 ± 0.098	0.066
Bl■	Blanes	41.6813°N	2.8147°E	32	4.81 ± 1.74	0.30 ± 0.20	0.429 ± 0.108	**0.161**
CC■	Cap de Creus	42.3172°N	3.3163°E	28	5.12 ± 1.07	0.64 ± 0.58	0.577 ± 0.083	**0.200**
Ml■	Mallorca	39.4168°N	3.2756°E	32	2.15 ± 0.37	0.29 ± 0.29	0.285 ± 0.080	0.084
Mr★	Marseille	43.2157°N	5.3408°E	30	3.88 ± 0.84	1.17 ± 0.38	0.431 ± 0.092	0.086
TT●	Tan Tan	28.6925°N	11.1120°W	30	5.63 ± 2.06	2.26 ± 1.91	0.545 ± 0.134	**0.128**
Tf●	Tenerife	28.3986°N	16.6449°W	48	2.10 ± 0.31	0.54 ± 0.39	0.304 ± 0.092	−0.040
PP★	Porto Palo	36.6855°N	15.1404°E	30	5.34 ± 1.48	0.77 ± 0.50	0.631 ± 0.067	**0.132**
Cr★	Crotone	38.9114°N	17.1716°E	23	4.10 ± 1.47	0.24 ± 0.24	0.446 ± 0.141	**0.146**
Sc★	Sciacata	37.4956°N	13.0199°E	25	3.27 ± 1.09	0.25 ± 0.16	0.322 ± 0.121	**0.185**
SE★	Sant’Elia	38.1896°N	13.3599°E	30	4.65 ± 1.13	0.03 ± 0.02	0.524 ± 0.125	0.027
TU★	Torre Uluzzo	40.1589°N	17.9546°E	25	2.15 ± 0.22	0.14 ± 0.14	0.259 ± 0.097	**−0.213**
Ot★	Otranto	40.0322°N	18.4534°E	23	2.38 ± 0.35	0.13 ± 0.12	0.249 ± 0.074	0.138
Mean				31 ± 1	5.02 ± 0.49	0.53 ± 0.08	0.51 ± 0.04	

Symbols following population code indicated morpho-species: circle - *C. tamariscifolia*; square - *C.mediterranea*; star - *C. amentacea*.

At each site throughout the geographical distribution, 30 to 48 individuals of each species were sampled. The minimum distance between sampled individuals was 0.5 m. A small clean piece of the apical branch was collected per individual, preserved in silica gel. Between two and four voucher specimens from most of the sampling sites were lodged in the herbarium of the University of Algarve (see Supplementary S1.4).

### Genetic data analysis

#### DNA extraction, microsatellite amplification and genotyping

DNA was isolated from 5–10 mg of dry tissue as in Zardi *et al*.^38^. Six microsatellite loci were amplified with fluorescently labelled primers as in Engelen *et al*.^27^. Fragment sizes were determined using an ABI PRISM 3130xl (Applied Biosystems) with GeneScan-500 (LIZ) as size standard. Alleles were manually scored using STRand^39^.

#### Genetic diversity and loci description

The number of alleles and private alleles was standardised by resampling for the smallest sample size (Od; N = 15) using HP-rare 1.1^40^. Nei’s unbiased gene diversity (expected heterozygosity) was obtained using GENALEX 6.502^41^. For comparisons between regions, data were standardised to a sample size of 23 individuals (excluding Od from analyses). The analyses were done for a minimum of 1 site per region since one of the clusters was just one location. One-way ANOVAs were performed to assess differences in the means of private alleles and allelic richness between regions, followed by Tukey tests for post hoc comparisons.

The analyses of departures from Hardy–Weinberg equilibrium (HWE) were estimated using GENALEX 6.502. The inbreeding coefficients (F_*IS*_) within each population were estimated with Genetix^42^ and tested with 10 000 permutations. MICRO-CHECKER v.2.2.3 software^43^ was used to check for scoring errors because of stuttering, large allele dropout and to estimate null allele frequencies. When evidence of null alleles was significant, a correction for null alleles was applied.

#### Population genetic structure

Genetic structure was inferred using STRUCTURE v2.4^44^ considering admixture and no *a priori* population assignment. The correlated allele frequency model was run with a burning time of 50000 repetitions, 500000 iterations and considering a range of clusters (K) from 1 to 31. The model was run 14 times for each *K* to check consistency. The number of clusters was estimated by Δ*K*(Evanno *et al*.^45^). A correspondence analysis based on individual allelic composition was performed using GENETIX^42^. All individuals with missing data at one or more loci were excluded (127 individuals). Finally, to assess isolation by distance (IBD), Mantel tests based on Jost’s D against the shortest sea distance were implemented in the R package vegan^46^. ANCOVA was performed to assess isolation by distance between clusters. To ensure independence between samples, we only considered populations separated by 150 to 700 km for the ANCOVA.

#### Rare hybridization vs. morphologically plastic species

To assess reproductive isolation between *C. amentacea* (Pi) and *C. tamariscifolia* (Ps) in parapatry (in “El Playazo”), STRUCTURE was used (as described above) to assign individuals to genetic groups and to detect putative admixed individuals. In this case, two genetic groups were considered (i.e. two taxa assumed). The mean of the posterior distribution of each individual admixture coefficient, q_x_^(i)^, represents the proportion of the i^th^ individual’s genotype drawn from cluster “x”. The concordance between the morphological and genetic classification was evaluated using the weighted kappa coefficient^47^, following the scale proposed by Monserud and Leemans^48^. Subsequently, STRUCTURE and kappa analysis were repeated (keeping K = 2) including the closest localities sampled where *C. tamariscifolia* (He) and *C. amentacea* (GV and Cq) occurred alone in the contact area to assess whether the individuals would group according to site or morphological entity.

## Results

### Distribution of *Cystoseira tamariscifolia*, *C. mediterranea* and *C. amentacea* through time

All distribution prediction models had good accuracy (Table 2). The current biogeographic distributions of *C. amentacea* and *C. mediterranea* were mainly explained by the minimum SST, while for *C. tamariscifolia* maximum and minimum SST had similar importance (Table 2). The models for the species complex yielded lower but still acceptable validation scores. The clamping mask did not detect uncertainty regions. The modelled distributions of *C. tamariscifolia* and the whole complex for the present time matched very well their known (empirical) distributions. For the Mediterranean endemic *C. amentacea* and *C. mediterranea*, the models predicted high probabilities of occurrence along the Atlantic coasts of the Iberian Peninsula and the Azores, with the North Atlantic African coast also suitable for *C. mediterranea* (Supplementary S1.2).

**Table 2:** Relative importance of the maximum (MaxSST) and minimum (MinSST) sea surface temperature, and mean validation scores (TSS - True Skill Statistics; AUC - Area Under the receiver operating characteristic Curve; Sens. - Sensitivity; Spec. - Specificity) for the ensemble of each taxa.

	MaxSST	MinSST	TSS	AUC	Sens	Spec
*C. amentacea*	0.114	0.875	0.81	0.95	93.27	87.8
*C. mediterranea*	0.174	0.873	0.76	0.92	97.5	78.92
*C. tamariscifolia*	0.77	0.739	0.75	0.93	93.33	82.01
Species group	0.48	0.838	0.64	0.87	88.38	75.71

**Figure 1:**
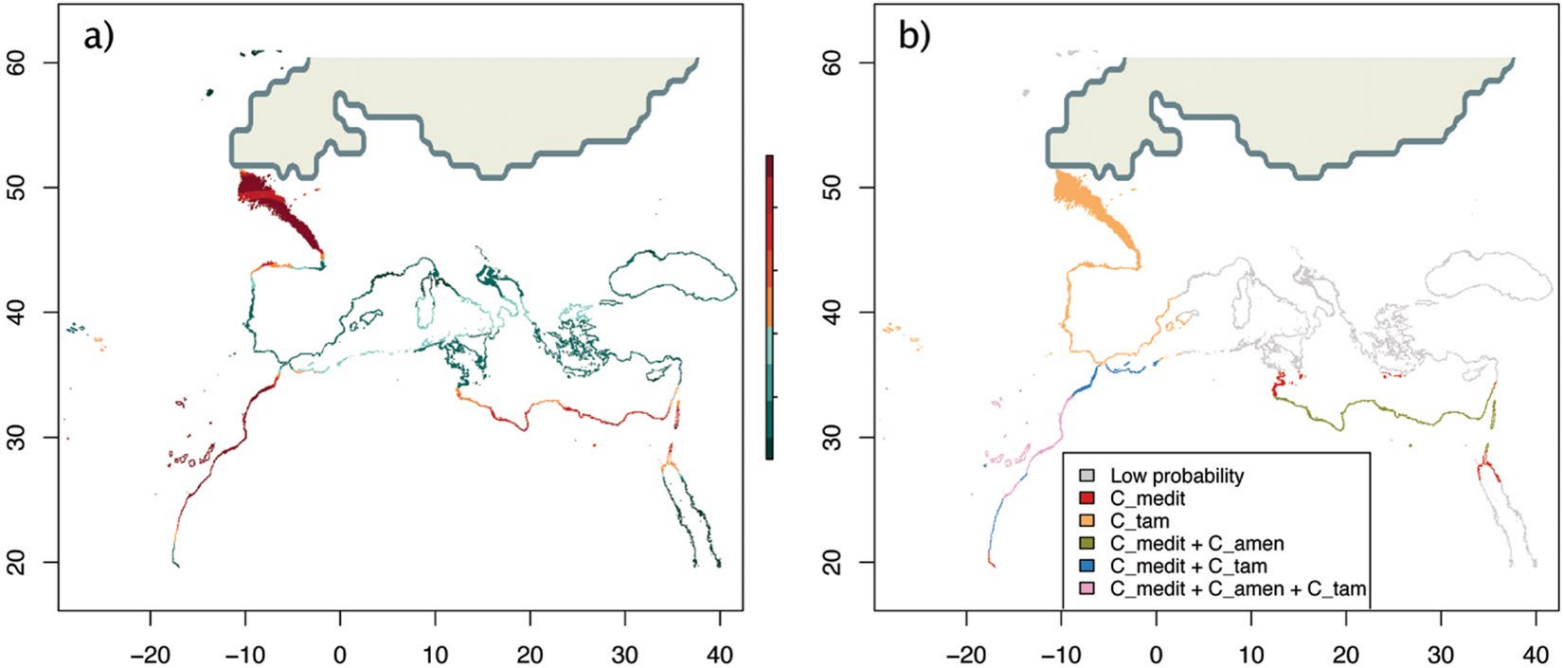
Last Glacial Maximum (LGM) hindcasts of *Cystoseira* showing the model obtained with the pooled occurrences of the three studied species (**a**) with probabilities ranging from 0 -dark blue- to 100 -dark red-), and the overlap of the models obtained for each species separately (**b**). High latitude LGM ice-sheet is depicted according to the oceanic CMIP5 reconstruction. (C_tam: *C. tamariscifolia*; C_amen: *C. amentacea*; C_medi: *C. mediterranea*).

The ensemble hindcast for the LGM suggested a past disjoint distribution of the species complex (Fig. 1a). Members of this complex could have persisted during the LGM in three main areas: SE Mediterranean, North Atlantic African coast, and the Celtic Sea. The latter seems an exclusive refugium for the putative *C. tamariscifolia* (Fig. 1b; Supplementary S1.3), which could have had a predicted continuous distribution range from 50°N to 20°N along European and North Atlantic African coasts and the West of the Western Mediterranean basin. According to the LGM models, *C. mediterranea* and *C. amentacea* could have persisted in the eastern coast of Libya and southern Sicily, whereas *C. tamariscifolia* could have persisted along Atlantic Iberian and Alboran coasts, as well as in the potential refugium of suitable habitat shared by the three species along North Atlantic Africa.

### Genetic diversity and loci description

The six microsatellite loci amplified a total of 226 different alleles in 1043 individuals of the three species studied, with a range of 31–65 alleles per locus. The standardised allelic richness per site ranged from 1.22 (FN) to 11.47 (CB) (Table 1). The standardised number of private alleles per locus ranged from 0 to 2.26 (TT). Two localities (Nj and TU) had significant heterozygote excess, while in contrast, 14 out of the 34 sites had significant heterozygote deficiency (Table 1). No evidence of large allele dropout or stuttering scoring errors was found, while null alleles were rare to uncommon (proportion per locus < 0.25).

### Population genetic structure

**Figure 2:**
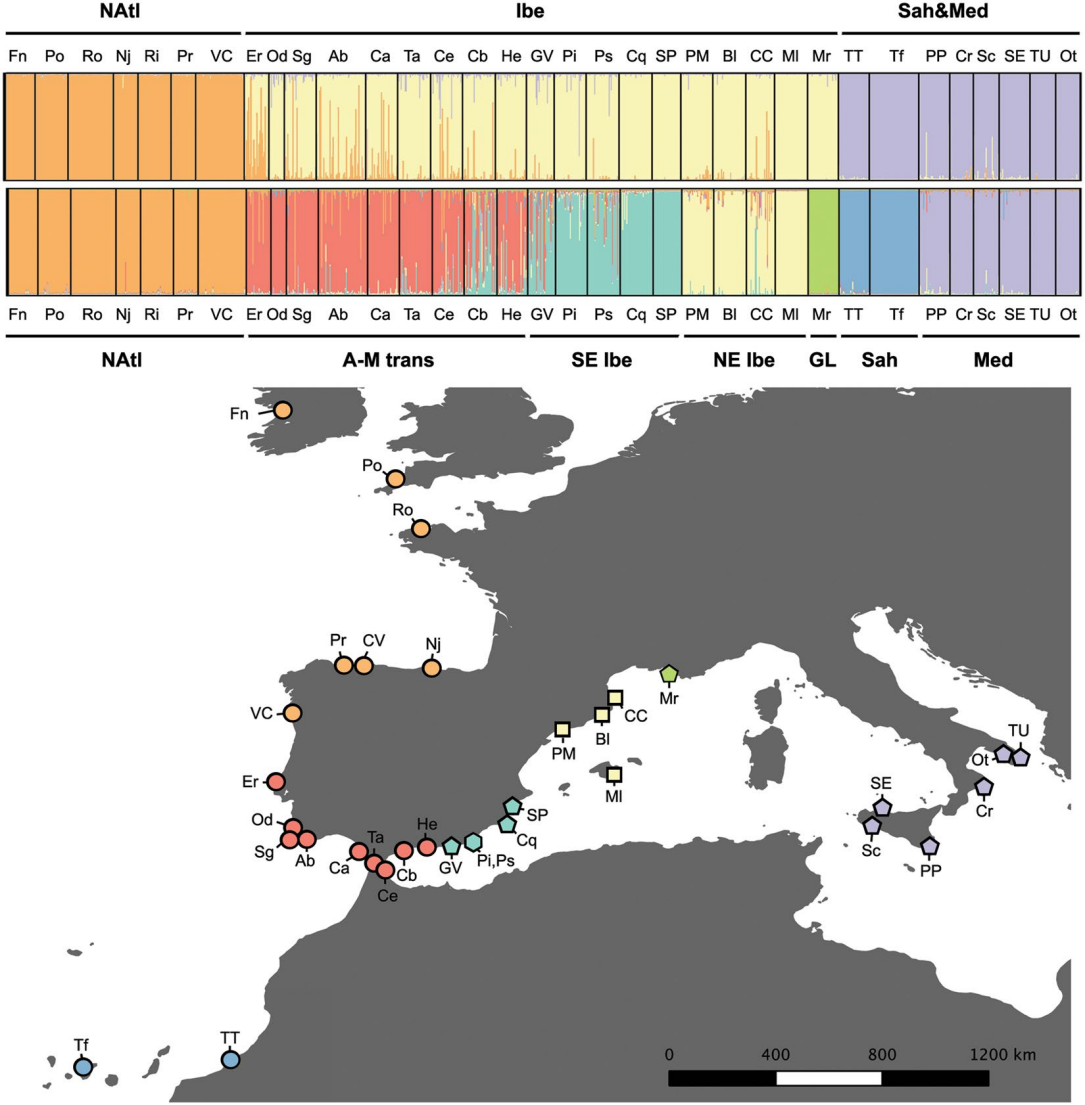
Population genetic structure of *Cystoseira* populations. (**a**) STRUCTURE assignment of individuals to 3 (upper plot) and 7 (lower plot) inferred clusters. A column represents each individual; different colours within columns indicate the maximum likelihood probability of belonging to different clusters. (**b**) Map of sampled localities coloured according to the dominant genetic cluster. Symbol indicates morpho-species: circle - *C. tamariscifolia*; square - *C. mediterranea*; pentagon - *C. amentacea*; and hexagon - parapatric populations of *C. tamariscifolia* and *C. amentacea*.

The estimated number of genetic clusters was *K* = 3, with a second maximum modal value for *K* = 7 (see Supplementary S3.1). However, K = 3 did not retrieve the three species, as individuals grouped more based on their geographical location rather than separating species (Fig. 2). For *K* = 3, sites were grouped as (1) North Atlantic (NAtl), including northern Portugal and all sites northwards, (2) Central Mediterranean and Saharan (CMed&Sah), the former including the southern Italy populations and the latter including the Canary and Saharan populations, and (3) Iberian (Ibe) including Iberian sites southwards from northern Portugal and the NW Mediterranean. For *K* = 7, the “CMed&Sah” cluster was divided into Central Mediterranean (CMed) and Saharan (Sah) populations; and the “Ibe” cluster was divided into four subclusters: Atlantic-Mediterranean transition (A-M trans), Southeastern Iberia (SEibe), Northeastern Iberia (NEibe), and Gulf of Lion (GL) (Fig. 2). It is noteworthy that in some runs with K = 3 the Saharan populations (i.e., TT and Tf) grouped with North Atlantic populations, and the NEibe (*C. mediterranea*) populations (i.e., PM, Bl, CC, and Ml) clustered with Central Mediterranean populations (data not shown).

**Figure 3:**
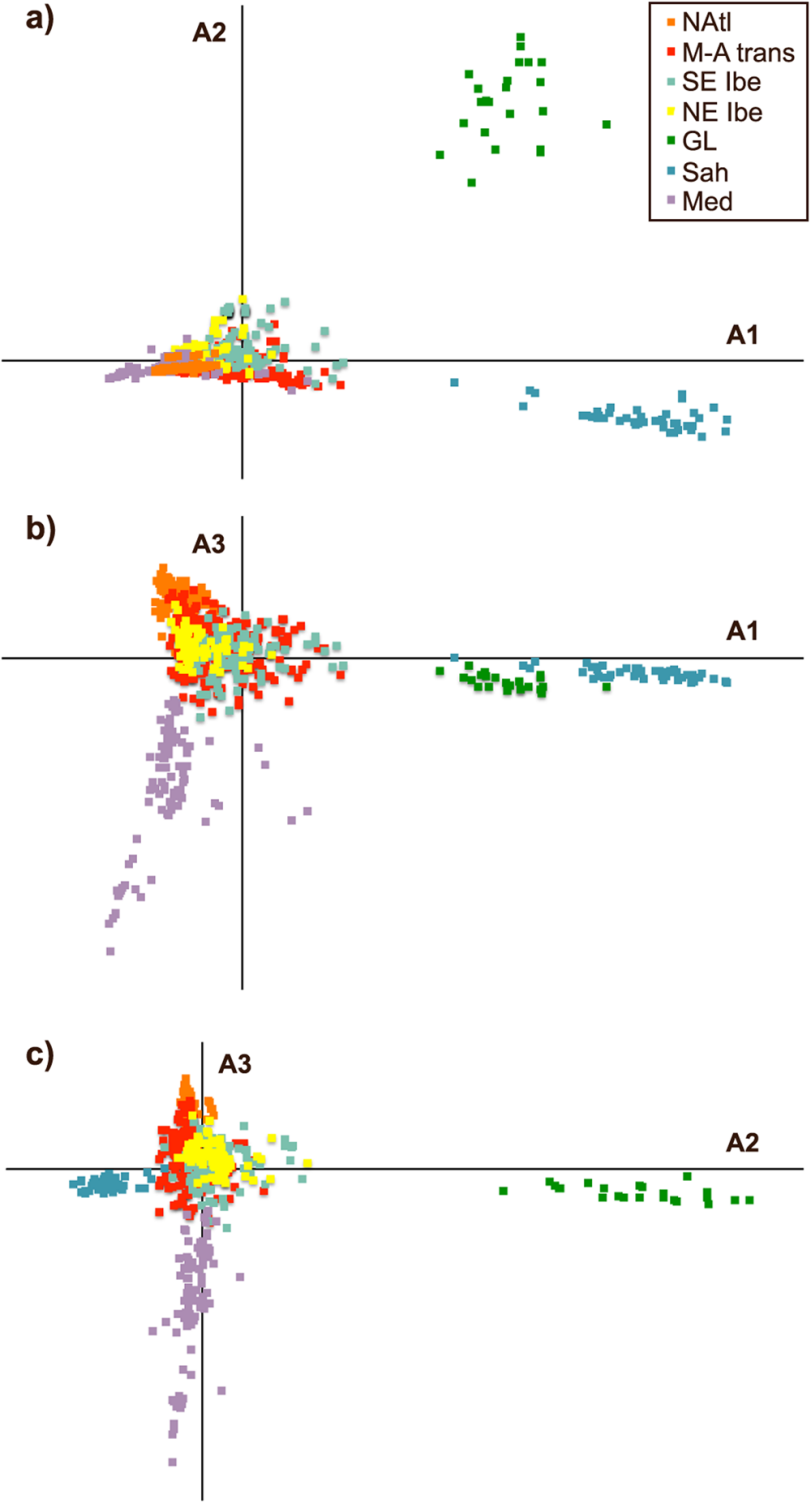
Correspondence Analysis based on allele composition for six microsatellites loci of *Cystoseira tamariscifolia, C. amentacea* and *C. mediterranea* individuals from different populations. A1 - Axis 1 (Inertia = 2.45%); A2 - Axis 2 (Inertia = 2.13%); A3 - Axis 3 (Inertia = 1.96%). Individuals coloured according to STRUCTURE results for k = 7 (Fig. 3).

The correspondence analysis explained a small part of total variability (6.54% in 3 axes; Fig. 3). The first axis discriminated Sah and GL (Fig. 3a,b), the second further distinguished GL (Fig. 3a,c) and the third separated CMed (Fig. 3b,c). Therefore, this analysis distinguished mainly four genetic groups (CMed, Sah, GL, and Iberian-NAtl) and indicated that GL and Sah populations were the most differentiated from the rest.

### Genetic diversity and isolation by distance between regions

**Figure 4:**
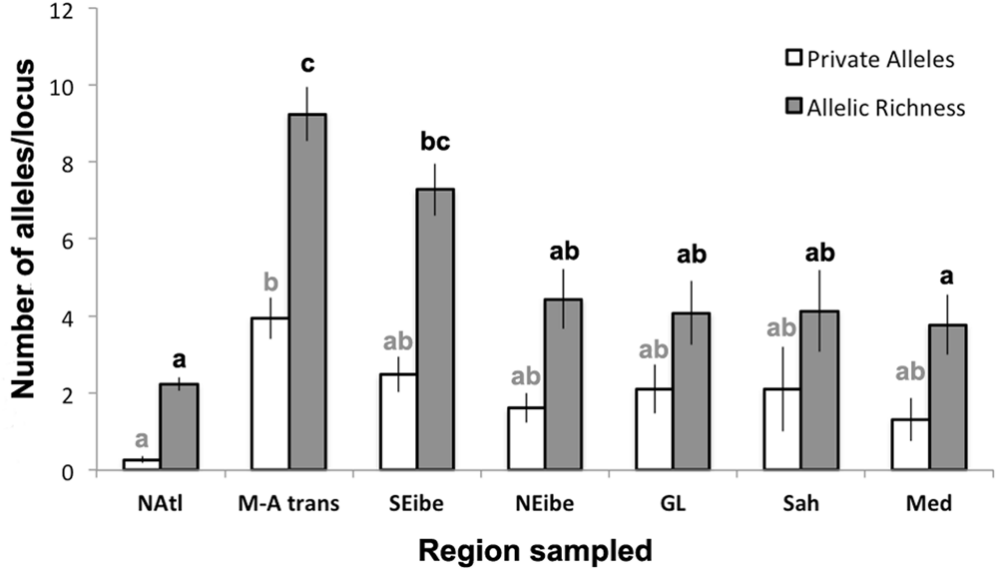
Standardised allelic richness (n = 23; pop = 1) and standardised private allele number for the seven identified subregions. Upper letters indicate the result of pairwise comparisons. Deviation bars represent the standard error.

The highest regional genetic diversity and number of private alleles within this complex were found in Iberia, mainly in the Atlantic-Mediterranean transition (from Er to He) and SE Iberia (from GV to SP) (Fig. 4). In contrast, the “NAtl” cluster showed the lowest allele richness and private alleles. The remaining clusters (i.e. GL, NEibe, CMed, Sah) showed similar and intermediate values of genetic diversity measures.

**Figure 5:**
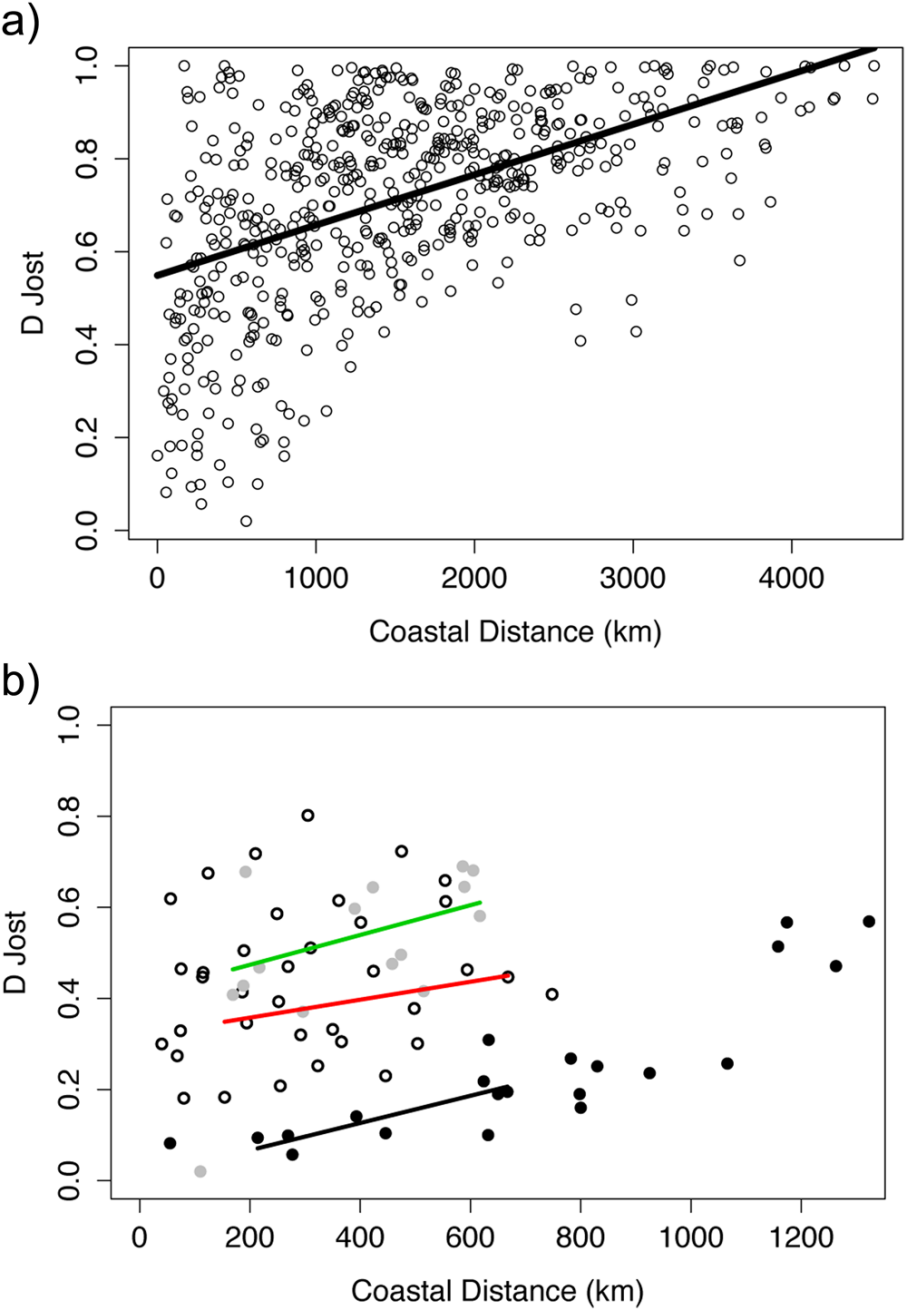
Relationship between Jost’s D genetic distance and the shortest sea distance considering: (**a**) all populations (mantel R = 0.507; p-value < 0.05); (**b**) only North Atlantic (black dots and black line; mantel R = 0.873; p-value < 0.05), Atlantic-Mediterranean transition (White dots and red line; mantel R = 0.157; p-value > 0.05) and Mediterranean populations (grey dots and green line; mantel R = 0.610; p-value < 0.05).

Jost’s D Genetic distance was significantly correlated with the shortest sea distance across all sampling sites (Fig. 5a), as well as in the Central Mediterranean and North Atlantic clusters, but not for the Atlantic-Mediterranean transition (Fig. 5b). The three regions studied separately showed a similar slope, but different intersection points, showing that, at similar inter-population distances, Northern Atlantic populations are the least differentiated, and Central Mediterranean populations the most differentiated (see Supplementary Table S4).

### Rare hybridization vs. morphologically plastic species

**Figure 6:**
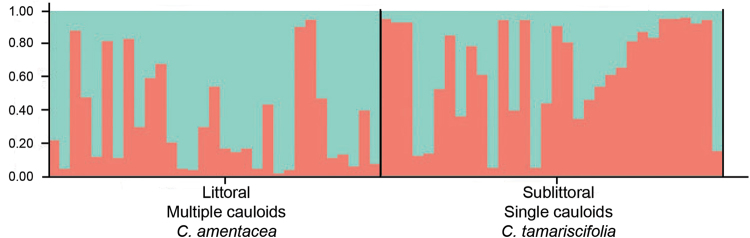
Proportion of ancestry of each sampled individual (columns) as inferred with STRUCTURE for six microsatellite loci in “El Playazo”.

The STRUCTURE analyses of *C. amentacea* and *C*. *tamariscifolia* (Fig. 6) at the parapatric contact zone of “El Playazo” showed many (approximately 31%) genetically intermediate individuals (admixture coefficients < 0.75) in both intertidal and subtidal habitats (χ^2^ = 0.036; p = 0.850). The kappa coefficient (0.545) indicated moderate agreement between genetic and morphological identification (Monserud and Leemans 1992). However, when other nearby localities (i.e., He, GV, and Cq) of *C. amentacea* and *C. tamariscifolia* were considered, the kappa coefficient decreased (kappa = 0.238) indicating poor agreement between morphological and genetic features. In the global analyses of the entire complex, these two parapatric populations were not genetically distinguishable (Fig. 2).

## Discussion

Our results show multiple, divergent and spatially structured genetic pools, which can be explained by past climate-driven range shifts. We further show that the morphological species traditionally recognized within this complex do not correspond with separate genetic groups and that those morphological species interbreed when co-occurring in contact zones, indicating that current taxonomical classification needs to be re-evaluated.

The STRUCTURE analyses revealed the existence of three main genetic clusters and seven spatially coherent sub-clusters. All clusters showed unique alleles and significant differentiation (Fig. 4; See Supplementary S3), also suggesting historical and ongoing barriers to gene flow among these regions. Indeed, our models hindcasted a disjoint potential distribution of the species complex during the LGM, split into three main regions – 1) NW Africa, 2) from Biscay Bay to the Celtic Sea in the Atlantic, and 3) SE Mediterranean. Coincidentally, the independent genetic evidence sampled in the present ranges also groups all the individuals of this complex into three main genetic clusters – 1) S Iberia and NW Mediterranean, 2) N Atlantic, and 3) a disjunct Central Mediterranean and Saharan cluster. The arrangement of some sub-clusters, notably Sah and NEIbe, was not stable within higher cluster across runs (Sah sometimes grouping with NAtl and NEIbe sometimes grouping with Central Mediterranean). Comparing past isolated ranges and present genetic groups permits us to postulate some hypotheses. The inferred LGM refugium from the Celtic Sea (NE Atlantic) to northern Iberia corresponds to the present distribution of the differentiated northern genetic group. Surprisingly, the two remaining areas with high probability of occurrence during the LGM (NW Africa and SE Mediterranean) do not include Southern Iberia where the highest diversity of the complex is found. This could hypothetically be an effect of range shifts during the recent warmer climates, allowing the (presumably highly diverse) northern African group to colonize Southern Iberia. The Central Mediterranean genetic group (CMed) may have been colonized from the third inferred refugium in the SE Mediterranean, where the species currently appears to have a rather marginal presence (Supplementary S1.1).

If the three described morphospecies were indeed constituted of three separate entities, they would have most likely overlapped in geographical space during the LGM according to our hindcasts. If so, the contact of these putative morpho-species would date back to (and likely pre-date) the LGM and continue afterwards as a consequence of the expansion of refugial populations into a more continuous distribution area. If they were not strongly reproductively isolated, then widespread admixture and introgression would be expected. This scenario cannot be ruled out as the cause for the observed genetic patterns, although there is little evidence for admixture and introgression of distinct genetic groups co-occurring together. On the contrary, the clustering of individuals in multiple geographical regions suggests that the recovered genetic structure is not primarily defined by morphological species assignments and that each regional genetic group is a distinct lineage with a common ancestor.

The North Atlantic cluster showed the lowest genetic diversity and singularity of all the regions considered. Such a pattern is often a consequence of consecutive bottlenecks due to founder events, and its high differentiation suggests that the separation of this group is not recent (see Supplementary S3.2). The most likely hypothesis for such a present scenario is a past postglacial colonization from a genetically distinct group, such as northern Atlantic Iberia, where the models indicate that an ancient refugial population could have persisted.

The southern Atlantic Iberian Peninsula (clusters Atlantic-Mediterranean transition and SE Iberia) hosted the highest genetic diversity. This follows expectations as this region is considered an important contact area where different morpho-entities meet^21,28,30^, and potentially hybridize^23,25,31^. However, the high differentiation there also suggests it could have been a stable LGM climate refugium. This hypothesis is supported by the *C. tamariscifolia* and *C. mediterranea* models (Fig. 1b), but not by the model for the species complex (Fig. 1a). Thus, the high genetic diversity and singularity in this area could be a consequence of a double role of southern Iberia as secondary contact area and as a climatic refugium. Furthermore, the high number of unique alleles found in this area could also have their origin in nearby, unsampled populations from northern Africa.

Saharan and Central Mediterranean populations, in comparison with other regions, showed many unique alleles and low allelic richness, a common genetic signature in rear-edges^3,49^ where ancient divergent populations are presently undergoing bottlenecks^50^. The models for the whole complex predicted a post-LGM retreat in the southernmost Atlantic distribution edge, supporting this hypothesis. In the Mediterranean, models suggested important range changes since the LGM and a current and past range edge location of Sicilian populations. Thus, the relatively low allelic richness found in Saharan and Mediterranean populations should be the consequence of genetic erosion associated with their marginal and unstable character. In contrast, the clusters Northeast Iberia and Gulf of Lion (one population only) showed similar allelic richness and number of unique alleles as Saharan and Central Mediterranean populations. However, the different models do not support long-term persistence there. Thus, the high number of private alleles could have their origin in other regions nearby that might presently be extinct or unsampled. Future studies of the genetic diversity and differentiation of extant Libyan populations will be key to better understand the phylogeography of this complex since according to the models these should be the most stable populations in the Mediterranean Sea.

Overall, high genetic structure was observed even at small spatial scales. Heterozygote deficits and high rates of inbreeding were also pervasive, especially in the Mediterranean Sea. This is expected considering the reproductive biology of these species as selfing hermaphrodites and it agrees with previous findings for populations of *C. amentacea* in the Gulf of Lion^51^ and Sicily^52^, and other fucoid algae^53^. Beyond the historical processes described above, patterns of genetic differentiation within and among populations are also determined by mating systems, dispersal processes and meta-population dynamics acting at different spatio-temporal scales^52^. The dispersal potential of eggs and embryos of the genus *Cystoseira* is low due to their negative buoyancy and because as gametes are released the eggs remain attached to the alga until settlement takes place later already as an embryo^54^. This promotes selfing, bi-parental inbreeding and spatial clustering of related individuals. Long-distance dispersal may, however, take place via detached and drifting fertile specimens^55^, a particularly relevant means for colonization of new habitats. Moreover, although settling embryos attach readily, wave action-mediated translocation of embryos on scales of hundreds of meters is conceivable^52^.

The higher genetic differentiation observed between geographically close populations, especially in the Mediterranean cluster, suggests important density barrier effects^56,57^, also known as priority colonization effects. Mediterranean populations of these *Cystoseira* spp. are restricted to the first centimetres of depth forming dense canopies and continuous belts throughout long stretches of coasts^28,55^. The saturation of available habitat is likely to favour local inbreeding and reduce the genetic impact of rare long-distance immigrants, i.e., contributing to the maintenance of differentiated gene-pools despite some migration and gene flow. By contrast, North Atlantic populations are usually composed of scattered individuals or patches appearing over wider areas and depth ranges^28,58^. The more important role of water motion and greater habitat diversity in the Atlantic, due to the interaction between wave exposure, tide and geomorphology^58,59^, should support a seascape with more frequent gaps favouring immigration and recolonization events. Furthermore, floating aerocysts that favour dispersal are more abundant and frequent in Atlantic (*C. tamariscifolia*) than in Mediterranean specimens (*C. mediterranea* and *C. amentacea*) of these *Cystoseira* species^28–30^. However, the hypothesis that Northern Atlantic populations could have originated from just one or a few Iberian refugia, while the Mediterranean could have been recolonised from different glacial refugia from Eastern Mediterranean, Iberian Peninsula and Northern Africa, might also partially explain these differences in genetic structure and similarity.

Our local-scale analyses revealed that when in parapatry (intertidal versus subtidal) the morphological entities *C. tamariscifolia* and *C. amentacea*, showed significant but low genetic differentiation, suggesting extensive admixture. The detection of hybridization between these entities was previously suggested based on intermediate morphological traits^24,25,31^. Since these parapatric populations of *C. tamariscifolia* and *C. amentacea* are more similar to each other than to other allopatric conspecific populations, there is no evidence to support their retention as distinct species. The poor match observed between morphological and genetic classification at such a local scale is best explained by the distinct environmental conditions experienced (affecting morphology) and by the lower connectivity between littoral and sublittoral subpopulations, rather than by reproductive barriers. It is therefore concluded that *C. amentacea* and *C. tamariscifolia* along the Alboran Sea should be considered a single entity, likely *C. tamariscifolia*. Hence, differences in morphotypes (i.e., number of cauloids) between different environmental conditions (littoral vs. sublittoral) and canopy features (dense meadow vs. scarce individuals) seem to be responses to the environment and may have poor taxonomic value. We hypothesize that intertidal stands are more exposed to surf and that their typical caespitose habit is a consequence of recurrent break of cauloids and primary branches, whereas more protected sublittoral stations allow the development of a main axis. Accordingly, previous studies on other fucoids identified marked morphological variations (e.g. branch length, number of main axes, or holdfast size) along depth, population density or wave exposure gradients^60,61^, as well as low connectivity between littoral and sublittoral specimens^62^.

Of the seven secondary clusters identified, populations from the Gulf of Lion and Sahara were the most differentiated (Fig. 3). In the case of Saharan populations, this differentiation is probably, at least partly, due to geographical distance. In the case of the Gulf of Lion, an important genetic break was observed between *C. mediterranea* from NE Iberia and *C. amentacea* from Marseille (“GL” cluster). These populations, separated by a relatively short geographical distance, are genetically isolated indicating either a reproductive barrier (i.e., two independent genetic entities), a dispersal barrier, or density barrier effects. In this sense, the existence of well-developed *C. mediterranea* belts in northeast Iberia^63^ and *C. amentacea* in the eastern area of the Gulf of Lion^55^ could preclude the expansion of immigrant genes by density barrier effects. This, in combination with long sandy coast and the Rhône Estuary hindering the arrival of putative immigrants, could explain this break. It is remarkable that this *C. amentacea* population (Gulf of Lion) was among the most differentiated populations, and that *C. mediterranea* (in the NE Iberian cluster) was genetically closer to *C. amentacea* from southern Spain and Sicily. The type locality for *C. amentacea* var. *stricta* is Algiers (Algeria), which is geographically closer to the southern Iberian and Sicilian populations of this species than GL. Thus, the existence of marked genetic divergence between *C. amentacea* from the Gulf of Lion and South-Western Mediterranean populations is expected and could have important taxonomical implications.

In summary: (i) the general clustering of individuals by geographical region rather than by current taxonomical identity, (ii) the existence of genetic clusters or subclusters that include populations assigned to *C. tamariscifolia*, *C. amentacea* and *C. mediterranea* (i.e., Iberia and SE Iberia) and (iii) the extensive gene flow between *C. tamariscifolia* and *C. amentacea* in the Alboran Sea all indicated that there is no support for three separate species within the *C. tamariscifolia* complex as currently accepted, and that the morphological characters traditionally used to distinguish among these species have poor taxonomic value. Specifically, our analyses of microsatellite data support the hypothesis that the *C. tamariscifolia* complex corresponds to a single, extremely polymorphic and highly structured species over the competing hypothesis that it corresponds to three well defined entities matching distinct gene pools and largely non-overlapping distributions. A single species also seems to be the most likely hypothesis considering previous studies pointing out the existence of intermediate morphologies that hamper accurate species determination^23,25,29,31^, so that identification often relies on the region of collection rather on the observation of specific and diagnostic morphological features. This view actually has some historical support. Accordingly, C. Agardh originally described *C. tamariscifolia* and *C. amentacea* as different varieties of the same specific entity^64^. If the three species are eventually considered a single taxonomic entity, *C. tamariscifolia*^21,65^ has nomenclatural priority and that name that should be retained. However, despite the obvious lack of support of microsatellite data for three separate species within the *C. tamariscifolia* complex, we do not consider this course of action at present as incomplete taxon sampling does not allow an unambiguous assignment, especially in the case of *C. amentacea*. In the case of *C. mediterranea* and *C. tamariscifolia*, populations close to the type localities were sampled (i.e. Cap de Creus −40 km from Banyuls sur Mer, Mediterranean France^31^; and Polzeath, Cornwall, England^65^ respectively), but not in the case of *C. amentacea*. Furthermore, different varieties of *C. amentacea* has been described based on material from different parts of the Mediterranean, such as Algier (Algeria; var. *stricta*)^66^, or Cap Tenare (Greece; var. *amentacea*)^64,67^, beside others varieties that have been suggested as synonyms from the Adriatic Sea (e.g. Dalmatian coast; var. *spicate* (Ercegovic) Giaccone; *Cystoseria spicate* Ercegovic)^68^, which make more difficult a final taxonomic assessment of this species complex. Furthermore, the Mediterranean taxa *C. amentacea* and *C. mediterranea* have longstanding and widespread use in ecological studies and their final synonymization with *C. tamariscifolia* s.s. would benefit from additional data before being fully adopted (e.g. independent nuclear sequence data, data from unsampled regions where cryptic sibling species may occur, such as the Adriatic). In the future our key inference for a single entity could be verified using nuclear multi-gene approaches taking into account divergence with gene-flow^69^ and reticulation, and by experiments determining reproductive compatibility among populations assigned to different genetic clusters^70^. Such studies may provide new insights and help capture with a finer resolution the historical patterns of diversification within this ecologically important complex.

## Electronic supplementary material

Supplementary info
