# Supplementary material for: Marine forests of the Mediterranean-Atlantic Cystoseira tamariscifolia complex show a southern Iberian genetic hotspot and no reproductive isolation in parapatry
Source: Sci Rep. 2018 Jul 11;8:10427. doi: 10.1038/s41598-018-28811-1 (PMC6041324; doi:10.1038/s41598-018-28811-1)
Supplement: Supplementary file 1 — Supplementary info [file 41598_2018_28811_MOESM1_ESM.pdf]

Marine forests of the Mediterranean-Atlantic *Cystoseira tamariscifolia* complex show a southern Iberian genetic hotspot and no reproductive isolation in parapatry

Ricardo Bermejo, Rosa M. Chefaoui, Aschwin H. Engelen, Roberto Buonomo, João Neiva, Joana Ferreira-Costa, Gareth A. Pearson, Núria Marbà, Carlos M. Duarte, Laura Airoldi, Ignacio Hernández, Michael D. Guiry, Ester A. Serrão.

Supplementary material:

Appendix S1 Supplementary data and results regarding SDMs and list of specimens deposited in the herbarium of the University of Algarve.
Appendix S2 Supplementary data and results regarding the parapatric populations of *C. amentacea* and *C. tamariscifolia* from "El Playazo".
Appendix S3 Supplementary results regarding genetic structure, diversity and differentiation.
Appendix S4 Supplementary results regarding genetic isolation by distance.

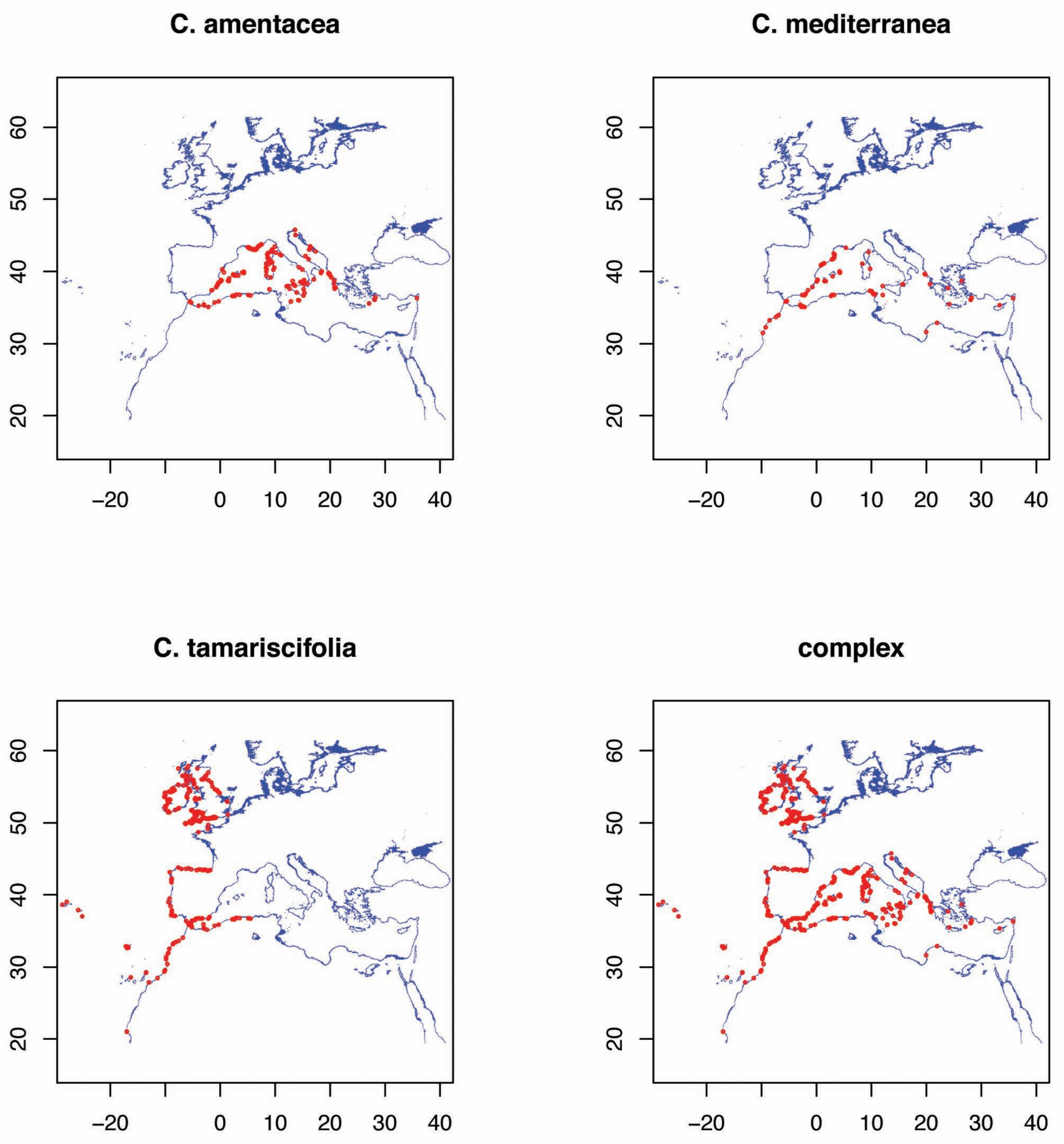

Supplementary S1.1. Georreferenced records used to build Spatial Distributuion Models of *Cystoseira amentacea, C. mediterranea, C. tamariscifolia* and *C. ericaefolia* complex.

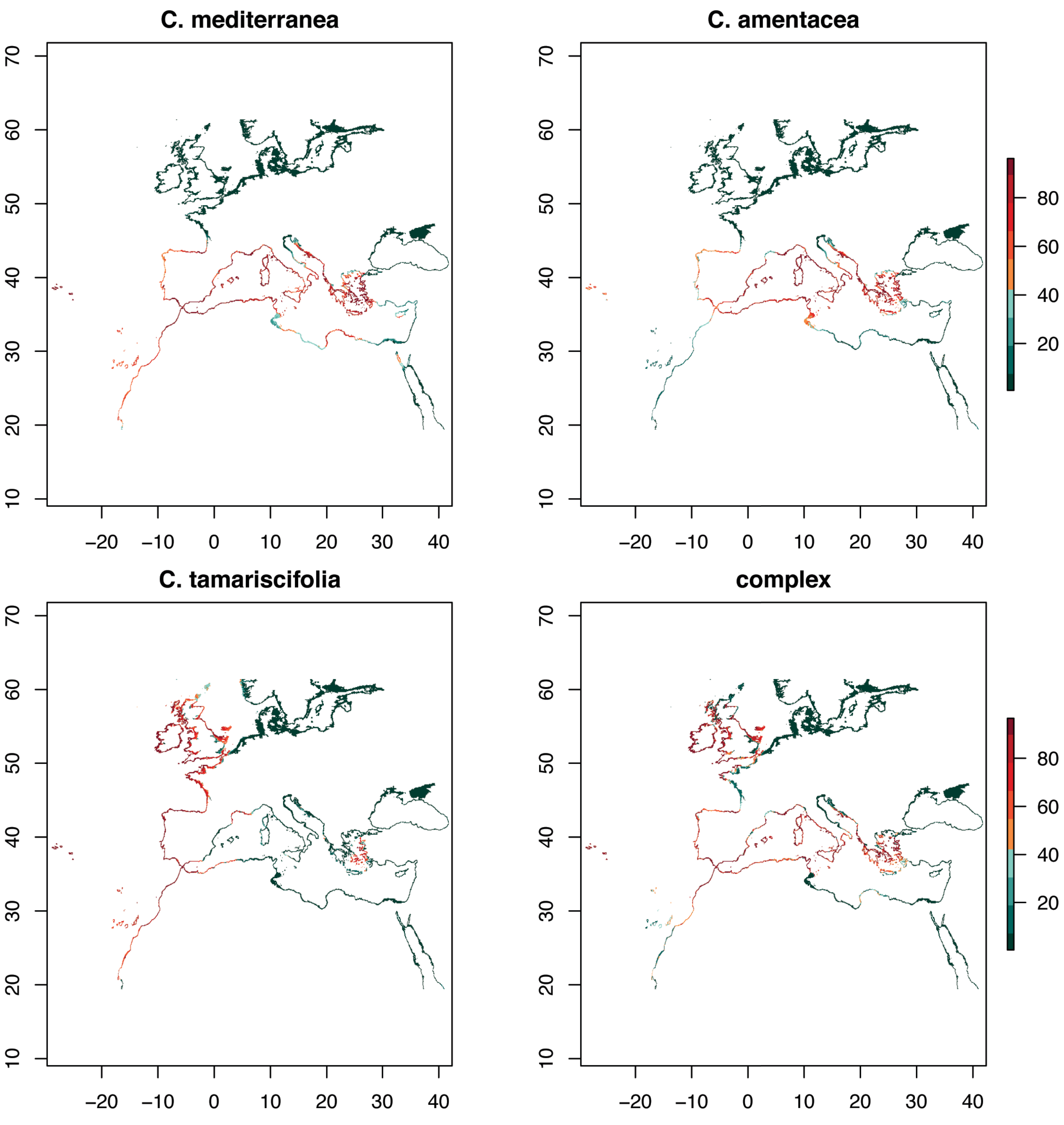

Supplementary S1.2. Assemble map of distribution of *Cystoseira amentacea, C. mediterranea, C. tamariscifolia* and *C. ericaefolia* complex for the present time using six presence-absence algorithms.

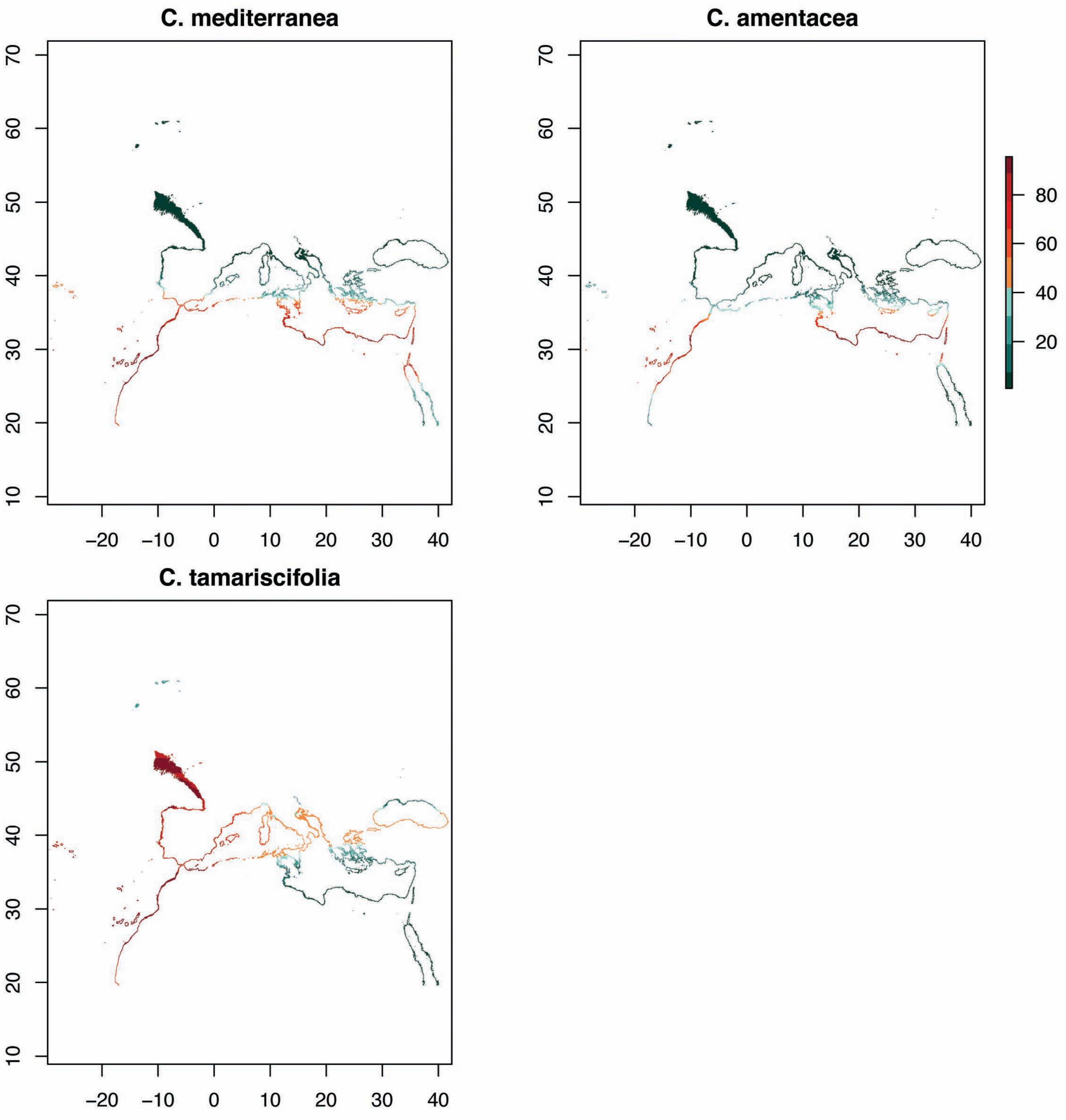

Supplementary S1.3. Ensemble map of distribution of *Cystoseira amentacea, C. mediterranea, C. tamariscifolia* and *C. ericaefolia* complex for the Last Clacial Maximum using six presence-absence algorithms.

Species	Region	Local	Code	Data	nº ALGU	Latitude	Longitude	Details
Cystoseira tamariscifolia	Cornwall	Polzeath	Po	30/6/11	A999	50.5858	4.882	2 samples in silica
Cystoseira tamariscifolia	Bretanha	Roscoff	Ro	28/7/10	A996	48.7294	4.0108	2 samples in silica
Cystoseira tamariscifolia	Cantabria	La Noja	Nj	11/4/11	A993	43.4962	3.5247	2 samples in silica
Cystoseira tamariscifolia	Asturias	Porcia	Pr	15/4/11	A989	43.5676	6.8754	2 samples in silica
Cystoseira tamariscifolia	Viana do Castelo	Praia Norte, Viana do Castelo	VC	28/4/10	A988	41.6993	8.8567	2 samples in silica
Cystoseira tamariscifolia	Alentejo	Odeceixe	Od	18/6/10	A992	37.4388	8.804	2 samples in silica
Cystoseira tamariscifolia	Algarve	Praia Manuel Lourenço, Albufeira	Ab	17/8/10	A995	37.0761	8.2769	2 samples in silica
Cystoseira tamariscifolia	Cádiz	El Chato, Cadiz	Ca	30/9/12	A994	36.477676	-6.264495	2 samples in silica
Cystoseira tamariscifolia	Cádiz	El Mirlo (Tarifa)	Ta	14/1/09	A972	36.0596	-5.7198	Herbarium sheet
Cystoseira tamariscifolia	Cádiz	Tarifa	Ta	14/1/09	A998	36.059609	-5.719784	2 samples in silica
Cystoseira tamariscifolia	Málaga	Calaburras	Cb	9/6/12	A973	36.5061	-4.6397	Herbarium sheet
Cystoseira tamariscifolia	Málaga	Calaburras	Cb	26/1/09	A987	36.5061	-4.6397	2 samples in silica
Cystoseira tamariscifolia	Granada	La Herradura	He	21/6/08	A974	36.7361	-3.7577	Herbarium sheet
Cystoseira tamariscifolia	Granada	La Herradura	He	22/6/12	A986	36.736105	-3.757676	2 samples in silica
Cystoseira amentacea	Almeria	Guardias Viejas	GV	23/6/08	A965	36.6950	-2.8496	Herbarium sheet
Cystoseira tamariscifolia	Almeria	El Playazo (Cabo de Gata)	Ps	4/8/12	A975	36.8593	-2.0032	Herbarium sheet
Cystoseira tamariscifolia	Almeria	El Playazo (Cabo de Gata)	Ps	3/8/08	A990	36.8593	-2.0032	2 samples in silica
Cystoseira amentacea	Almeria	El Playazo (Cabo de Gata)	Pi	4/8/12	A966	36.8593	-2.0032	Herbarium sheet
Cystoseira amentacea	Almeria	El Playazo (Cabo de Gata)	Pi	3/8/08	A976	36.8593	-2.0032	2 samples in silica
Cystoseira amentacea	Alicante	Santa Pola	SP	27/1/13	A977	38.196482	-0.514723	2 samples in silica
Cystoseira mediterranea	Tarragona	Punta de La Mora	PM	20/11/12	A982	41.126227	1.344316	2 samples in silica
Cystoseira mediterranea	Girona	Cala Frances, Blanes	Bl	20/12/12	A983	41.681354	2.814728	2 samples in silica
Cystoseira mediterranea	Girona	Cap de Creus	CC	30/7/10	A985	42.317225	3.31634	1 sample in silica
Cystoseira mediterranea	Baleares	Porto Colom, Mallorca	Ml	2010	A984	39.416826	3.275612	2 samples in silica
Cystoseira amentacea	Sicily	Altavilla (north Sicily), Sant'Elia	SE	2013	A980	38.046146	13.559728	5 samples in silica
Cystoseira amentacea	Sicily	Mazara del Vallo (south Sicily)	Close SC	2013	A981	37.651799	12.577311	2 samples in silica
Cystoseira amentacea	Calabria	Crotone (Calabria)	Cr	2013	A979	39.093961	17.112076	2 samples in silica
Cystoseira amentacea	Puglia	Otranto (Puglia)	Ot	2013	A978	40.0322	18.4534	3 samples in silica

Supplementary S1.4. List of specimens deposited in the herbarium of the University of Algarve.

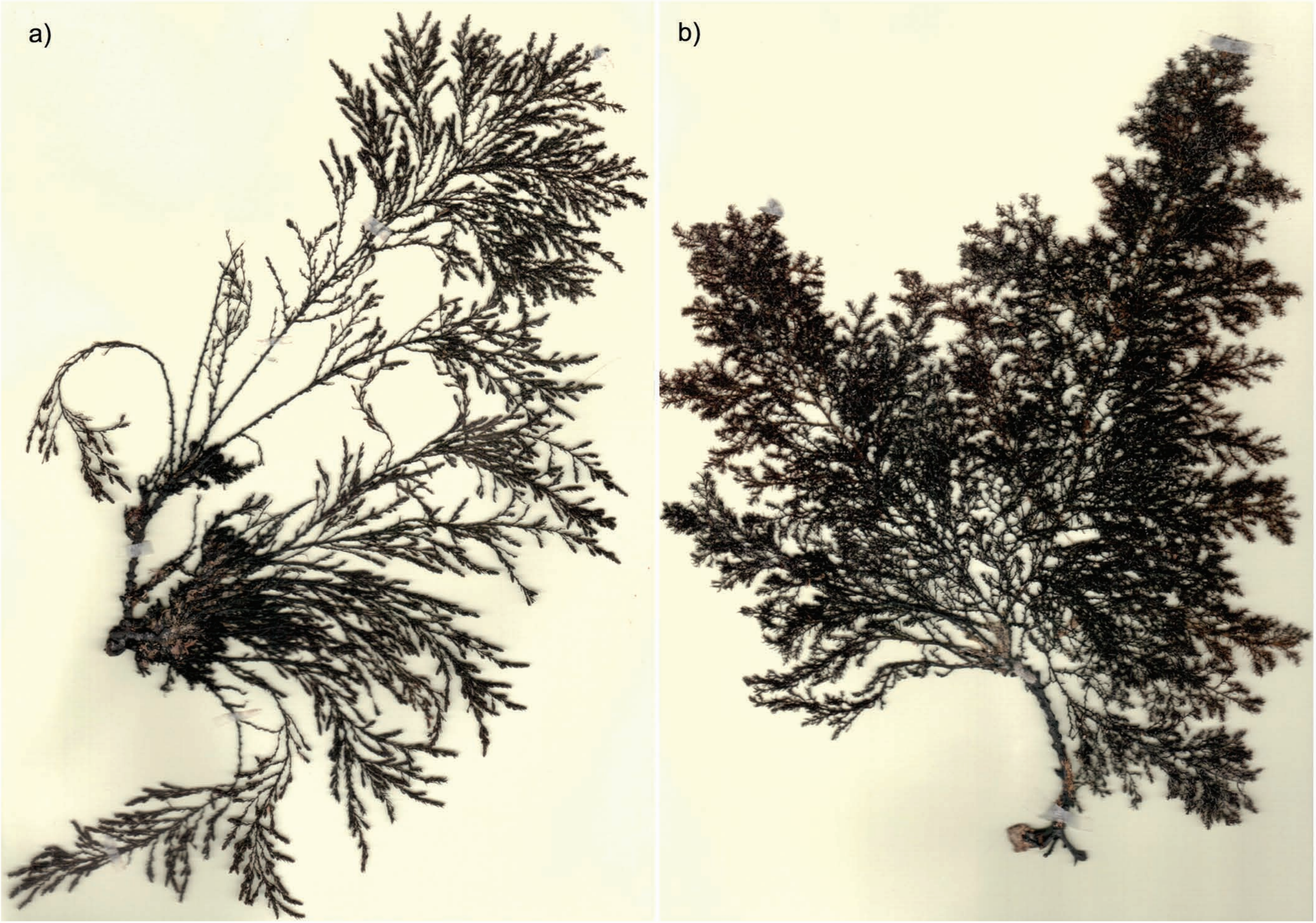

Supplementary S2. Habit of putative specimens of *Cystoseira amentacea* (a) and *C. tamariscifolia* (b) from “El Playazo”

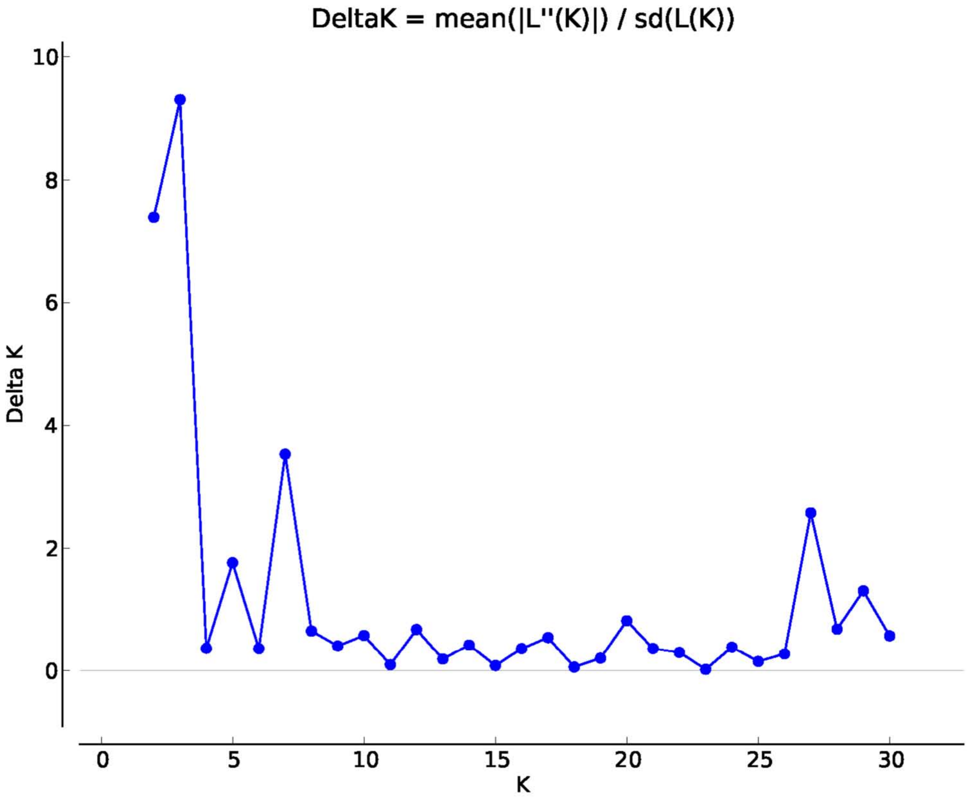

Supplementary S3.1. Plot of ΔK as a function of the number of cluster (K) across the 14 runs.

	FN	PO	RO	Nj	Ri	Pr	VC	Er	Od	Sg	Alb	Ch	Ta	Ce	CB	He	GV	PL	PS	Mu	SP	PM	BL	CC	Mal	Mar	TT	TF	PP	Cr	Sc	SE	TU	Ot
FN	0.00	0.31	0.27	0.47	0.57	0.51	0.57	0.72	0.88	0.64	0.69	0.87	0.89	0.74	0.85	0.74	0.85	0.92	0.81	0.97	0.84	0.91	0.79	0.73	1.00	0.99	0.90	1.00	0.82	0.71	0.93	1.00	0.82	0.83
PO	0.75	0.00	0.06	0.25	0.19	0.16	0.26	0.47	0.81	0.65	0.49	0.51	0.83	0.65	0.69	0.66	0.71	0.75	0.76	0.88	0.94	0.69	0.69	0.65	0.78	0.98	0.89	1.00	0.81	0.76	0.93	0.93	0.88	0.88
RO	0.63	0.19	0.00	0.19	0.22	0.10	0.24	0.42	0.79	0.60	0.53	0.55	0.83	0.65	0.67	0.63	0.74	0.74	0.75	0.88	0.91	0.67	0.69	0.65	0.88	0.93	0.88	0.93	0.79	0.68	0.91	1.00	0.82	0.83
Nj	0.59	0.35	0.28	0.00	0.09	0.10	0.19	0.39	0.78	0.60	0.49	0.43	0.73	0.51	0.58	0.60	0.70	0.65	0.63	0.83	0.88	0.65	0.79	0.65	0.86	0.88	0.93	0.87	0.87	0.78	0.83	0.99	0.78	0.79
Ri	0.69	0.34	0.35	0.11	0.00	0.08	0.10	0.41	0.81	0.70	0.47	0.35	0.75	0.64	0.62	0.70	0.70	0.67	0.72	0.83	0.95	0.58	0.74	0.62	0.81	0.95	0.95	0.96	0.89	0.83	0.88	1.00	0.89	0.90
Pr	0.70	0.32	0.21	0.11	0.12	0.00	0.14	0.32	0.77	0.60	0.45	0.40	0.79	0.61	0.55	0.63	0.65	0.59	0.68	0.80	0.92	0.53	0.68	0.62	0.80	0.87	0.90	0.88	0.82	0.71	0.87	0.99	0.83	0.84
VC	0.80	0.54	0.48	0.30	0.21	0.28	0.00	0.51	0.87	0.79	0.62	0.51	0.86	0.74	0.67	0.80	0.79	0.73	0.80	0.84	0.96	0.61	0.83	0.77	0.82	0.93	0.99	0.93	0.95	0.79	0.91	0.99	0.83	0.84
Er	0.63	0.45	0.42	0.24	0.30	0.25	0.47	0.00	0.72	0.21	0.25	0.38	0.66	0.46	0.45	0.41	0.46	0.61	0.60	0.76	0.73	0.56	0.66	0.61	0.75	0.98	0.86	0.97	0.74	0.74	0.80	0.95	0.79	0.79
Od	0.74	0.63	0.61	0.40	0.48	0.47	0.64	0.32	0.00	0.62	0.67	0.80	0.61	0.57	0.72	0.61	0.67	0.79	0.77	0.59	0.95	0.93	0.87	0.83	0.97	0.92	0.80	0.78	0.76	0.81	0.85	0.79	0.88	0.87
Sg	0.52	0.46	0.43	0.27	0.37	0.33	0.52	0.09	0.24	0.00	0.27	0.59	0.51	0.33	0.46	0.30	0.41	0.65	0.50	0.78	0.67	0.76	0.70	0.62	0.79	0.97	0.81	0.95	0.62	0.66	0.77	0.82	0.75	0.76
Alb	0.50	0.36	0.38	0.23	0.27	0.26	0.43	0.11	0.26	0.10	0.00	0.41	0.39	0.32	0.31	0.23	0.32	0.44	0.42	0.63	0.76	0.62	0.57	0.48	0.53	0.97	0.75	0.90	0.72	0.83	0.80	0.85	0.94	0.93
Ch	0.62	0.42	0.43	0.23	0.24	0.26	0.43	0.18	0.32	0.21	0.16	0.00	0.46	0.46	0.50	0.47	0.63	0.64	0.71	0.85	0.92	0.50	0.83	0.76	0.67	0.98	0.93	0.99	0.80	0.84	0.89	0.88	0.93	0.93
Ta	0.56	0.48	0.48	0.28	0.35	0.35	0.50	0.22	0.21	0.14	0.12	0.15	0.00	0.30	0.45	0.35	0.54	0.55	0.47	0.54	0.83	0.68	0.75	0.64	0.64	0.98	0.86	0.94	0.70	0.80	0.67	0.77	0.86	0.85
Ce	0.49	0.40	0.39	0.19	0.29	0.27	0.44	0.14	0.17	0.09	0.09	0.13	0.06	0.00	0.33	0.18	0.43	0.51	0.41	0.68	0.82	0.78	0.80	0.70	0.76	0.91	0.84	0.89	0.66	0.74	0.76	0.76	0.79	0.79
CB	0.52	0.40	0.40	0.21	0.28	0.25	0.41	0.14	0.21	0.11	0.08	0.14	0.09	0.06	0.00	0.18	0.25	0.16	0.18	0.50	0.71	0.46	0.59	0.49	0.60	0.87	0.82	0.87	0.64	0.76	0.67	0.77	0.90	0.89
He	0.50	0.41	0.40	0.23	0.32	0.29	0.47	0.14	0.19	0.08	0.07	0.14	0.08	0.04	0.03	0.00	0.37	0.41	0.30	0.67	0.73	0.66	0.66	0.57	0.69	0.91	0.76	0.85	0.58	0.71	0.75	0.78	0.86	0.85
GV	0.56	0.45	0.45	0.26	0.33	0.30	0.48	0.16	0.21	0.11	0.09	0.18	0.12	0.08	0.05	0.08	0.00	0.26	0.28	0.57	0.70	0.58	0.65	0.57	0.67	0.89	0.77	0.80	0.63	0.82	0.74	0.77	0.94	0.93
PL	0.57	0.46	0.45	0.26	0.33	0.29	0.46	0.21	0.25	0.18	0.13	0.19	0.13	0.10	0.03	0.09	0.06	0.00	0.16	0.46	0.76	0.47	0.61	0.50	0.55	0.84	0.83	0.81	0.67	0.79	0.64	0.81	0.92	0.91
PS	0.52	0.45	0.44	0.24	0.33	0.31	0.47	0.19	0.23	0.14	0.12	0.20	0.10	0.08	0.04	0.06	0.06	0.04	0.00	0.51	0.57	0.68	0.64	0.48	0.68	0.81	0.83	0.84	0.65	0.73	0.57	0.78	0.88	0.87
Mu	0.63	0.54	0.54	0.35	0.42	0.40	0.54	0.29	0.24	0.25	0.21	0.29	0.16	0.17	0.13	0.18	0.16	0.14	0.15	0.00	0.71	0.62	0.62	0.53	0.67	0.91	0.95	0.91	0.86	0.92	0.66	0.91	0.97	0.96
SP	0.91	0.84	0.80	0.66	0.72	0.73	0.82	0.56	0.67	0.47	0.47	0.57	0.48	0.45	0.41	0.43	0.44	0.46	0.38	0.49	0.00	0.80	0.79	0.71	0.84	0.84	0.97	0.97	0.91	0.99	0.78	0.93	0.98	0.96
PM	0.67	0.53	0.52	0.35	0.38	0.36	0.51	0.27	0.39	0.29	0.25	0.23	0.23	0.23	0.15	0.21	0.20	0.17	0.22	0.26	0.57	0.00	0.49	0.47	0.59	0.98	0.94	0.99	0.70	0.89	0.79	0.91	0.92	0.91
BL	0.72	0.62	0.61	0.48	0.52	0.50	0.66	0.38	0.47	0.34	0.29	0.40	0.32	0.31	0.25	0.28	0.29	0.28	0.28	0.33	0.65	0.32	0.00	0.12	0.73	0.93	0.86	0.99	0.66	0.90	0.71	0.86	0.95	0.95
CC	0.64	0.54	0.53	0.35	0.41	0.40	0.58	0.29	0.37	0.25	0.21	0.31	0.22	0.22	0.16	0.19	0.20	0.18	0.17	0.23	0.56	0.25	0.11	0.00	0.71	0.93	0.87	1.00	0.61	0.82	0.53	0.87	0.94	0.94
Mal	0.84	0.73	0.73	0.58	0.62	0.62	0.73	0.49	0.59	0.44	0.33	0.42	0.35	0.37	0.31	0.36	0.37	0.32	0.36	0.41	0.75	0.43	0.57	0.49	0.00	0.99	0.91	0.99	0.90	0.94	0.81	0.83	0.98	0.97
Mar	0.77	0.70	0.68	0.50	0.58	0.56	0.69	0.48	0.48	0.42	0.41	0.44	0.38	0.34	0.32	0.35	0.36	0.35	0.33	0.41	0.67	0.48	0.55	0.48	0.64	0.00	0.89	0.77	0.96	0.99	0.99	0.97	0.99	1.00
TT	0.69	0.62	0.61	0.46	0.53	0.51	0.65	0.39	0.38	0.32	0.30	0.37	0.30	0.27	0.26	0.26	0.27	0.29	0.28	0.37	0.65	0.42	0.47	0.40	0.56	0.48	0.00	0.49	0.87	0.95	0.97	0.94	1.00	1.00
TF	0.81	0.75	0.73	0.58	0.65	0.64	0.73	0.57	0.54	0.50	0.47	0.53	0.46	0.43	0.41	0.43	0.43	0.43	0.43	0.50	0.76	0.57	0.64	0.58	0.70	0.58	0.42	0.00	0.98	1.00	1.00	0.94	1.00	1.00
PP	0.65	0.57	0.56	0.41	0.49	0.46	0.62	0.32	0.34	0.24	0.27	0.31	0.23	0.20	0.19	0.19	0.20	0.23	0.21	0.32	0.60	0.32	0.38	0.30	0.53	0.47	0.40	0.56	0.00	0.37	0.47	0.60	0.48	0.50
Cr	0.74	0.66	0.62	0.47	0.55	0.51	0.66	0.40	0.44	0.32	0.36	0.40	0.32	0.28	0.28	0.28	0.32	0.32	0.29	0.41	0.73	0.45	0.54	0.44	0.63	0.56	0.49	0.65	0.25	0.00	0.42	0.64	0.41	0.43
Sc	0.83	0.75	0.73	0.54	0.62	0.62	0.73	0.48	0.52	0.41	0.40	0.47	0.33	0.34	0.31	0.35	0.36	0.33	0.29	0.38	0.73	0.47	0.53	0.39	0.65	0.62	0.55	0.69	0.35	0.41	0.00	0.68	0.69	0.68
SE	0.73	0.64	0.65	0.48	0.55	0.54	0.66	0.42	0.39	0.33	0.34	0.37	0.29	0.26	0.26	0.27	0.28	0.30	0.28	0.37	0.65	0.42	0.49	0.41	0.55	0.51	0.45	0.59	0.32	0.41	0.47	0.00	0.64	0.58
TU	0.89	0.81	0.77	0.61	0.69	0.69	0.78	0.55	0.62	0.47	0.50	0.54	0.46	0.41	0.44	0.45	0.49	0.48	0.46	0.54	0.84	0.58	0.67	0.60	0.76	0.69	0.63	0.75	0.42	0.49	0.68	0.53	0.00	0.02
Ot	0.90	0.82	0.77	0.61	0.69	0.69	0.79	0.55	0.62	0.46	0.49	0.54	0.45	0.41	0.43	0.44	0.48	0.47	0.45	0.54	0.85	0.58	0.67	0.60	0.76	0.69	0.62	0.75	0.43	0.50	0.67	0.50	0.09	0.00

Supplementary S3.2. Pairwise FST (lower left) and Jost's D (upper right) values of genetic distance.

Supplementary S3.3. Allele presence and frequency for the six loci of *Cystoseira* spp. populations analysed here. Sites are arranged by geographical clusters identify with STRUCTURE at K=7. For each site, a circle indicates that the corresponding allele was present; its diameter represents the frequency of that allele in the sample. These results were obtained using the MsatAllele R package (http://CRAN.R-project.org/package=MsatAllele)

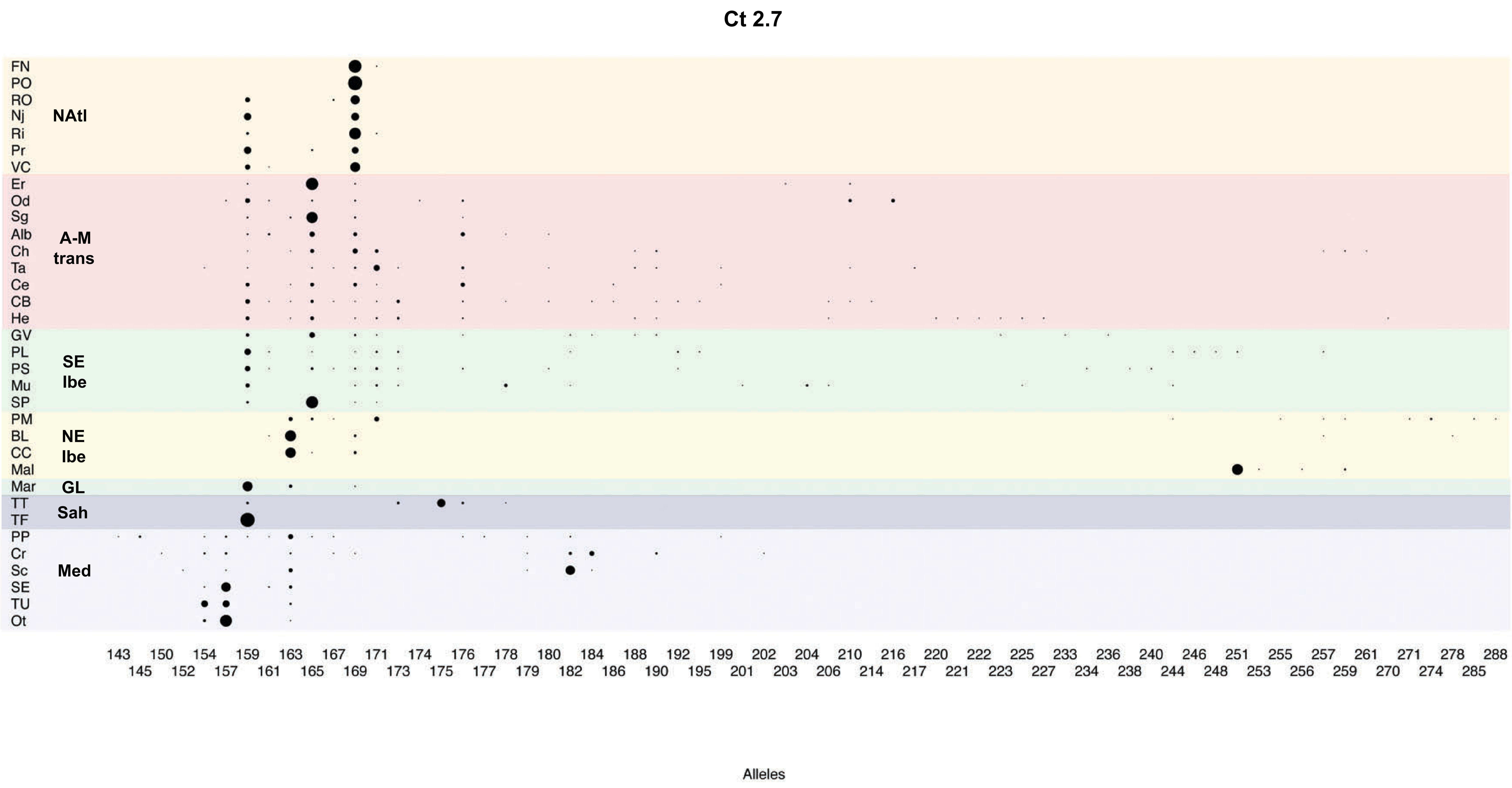

Ct 2.7
FN
PO
RO
Nj
Ri
Pr
VC
NAtl
Er
Od
Sg
Alb
Ch
Ta
Ce
CB
He
A-M
trans
GV
PL
PS
Mu
SP
SE
Ibe
PM
BL
CC
Mal
NE
Ibe
Mar
GL
TT
TF
Sah
PP
Cr
Sc
SE
TU
Ot
Med
143 145 150 152 154 157 159 161 163 165 167 169 171 173 174 175 176 177 178 179 180 182 184 186 188 190 192 195 199 201 202 203 204 206 210 214 216 217 220 221 222 223 225 227 233 234 236 238 240 244 246 248 251 253 255 256 257 259 261 270 271 274 278 285 288
Alleles

Ct 2.8

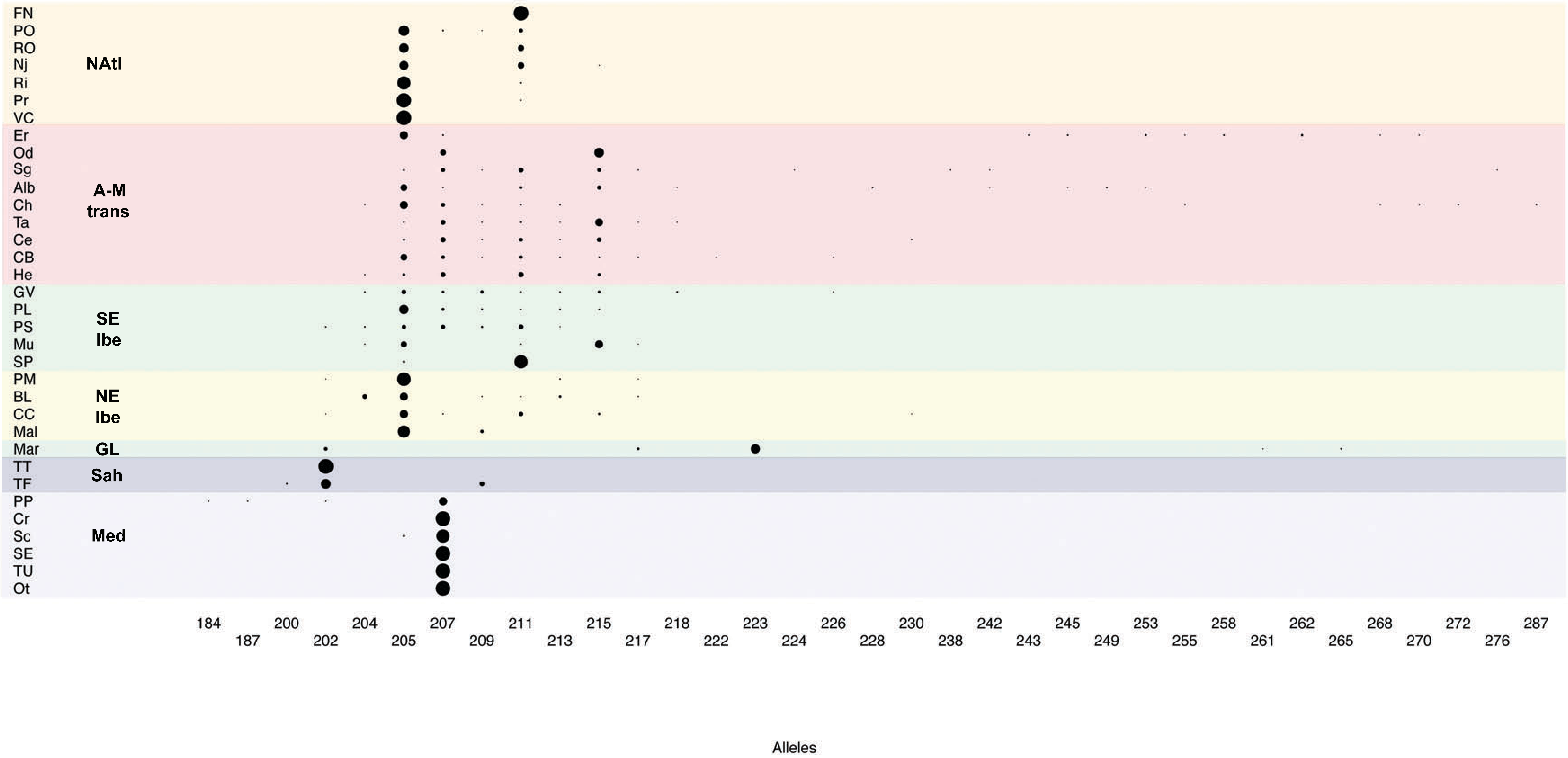
FN
PO
RO
Nj
Ri
Pr
VC
NAtl
Er
Od
Sg
Alb
Ch
Ta
Ce
CB
He
A-M trans
GV
PL
PS
Mu
SP
SE Ibe
PM
BL
CC
Mal
NE Ibe
Mar
GL
TT
TF
Sah
PP
Cr
Sc
SE
TU
Ot
Med
184 187 200 202 204 205 207 209 211 213 215 217 218 222 223 224 226 228 230 238 242 243 245 249 253 255 258 261 262 265 268 270 272 276 287
Alleles

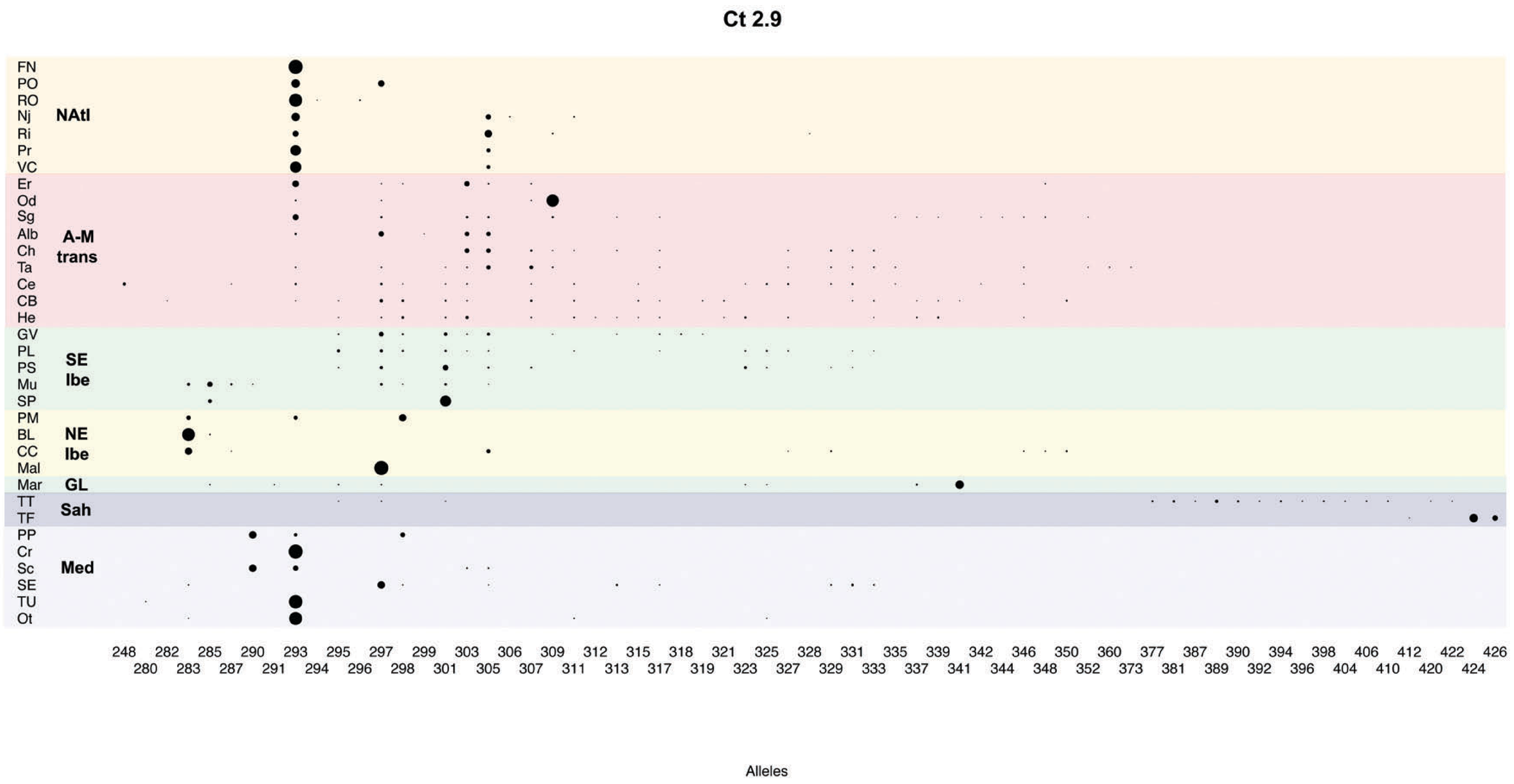
Ct 2.9
FN
PO
RO
Nj
Ri
Pr
VC
NAtl
Er
Od
Sg
Alb
Ch
Ta
Ce
CB
He
A-M trans
GV
PL
PS
Mu
SP
SE Ibe
PM
BL
CC
Mal
NE Ibe
Mar
GL
TT
TF
Sah
PP
Cr
Sc
SE
TU
Ot
Med
248 280 282 283 285 287 290 291 293 294 295 296 297 298 299 301 303 305 306 307 309 311 312 313 315 317 318 319 321 323 325 327 328 329 331 333 335 337 339 341 342 344 346 348 350 352 360 373 377 381 387 389 390 392 394 396 398 404 406 410 412 420 422 424 426
Alleles

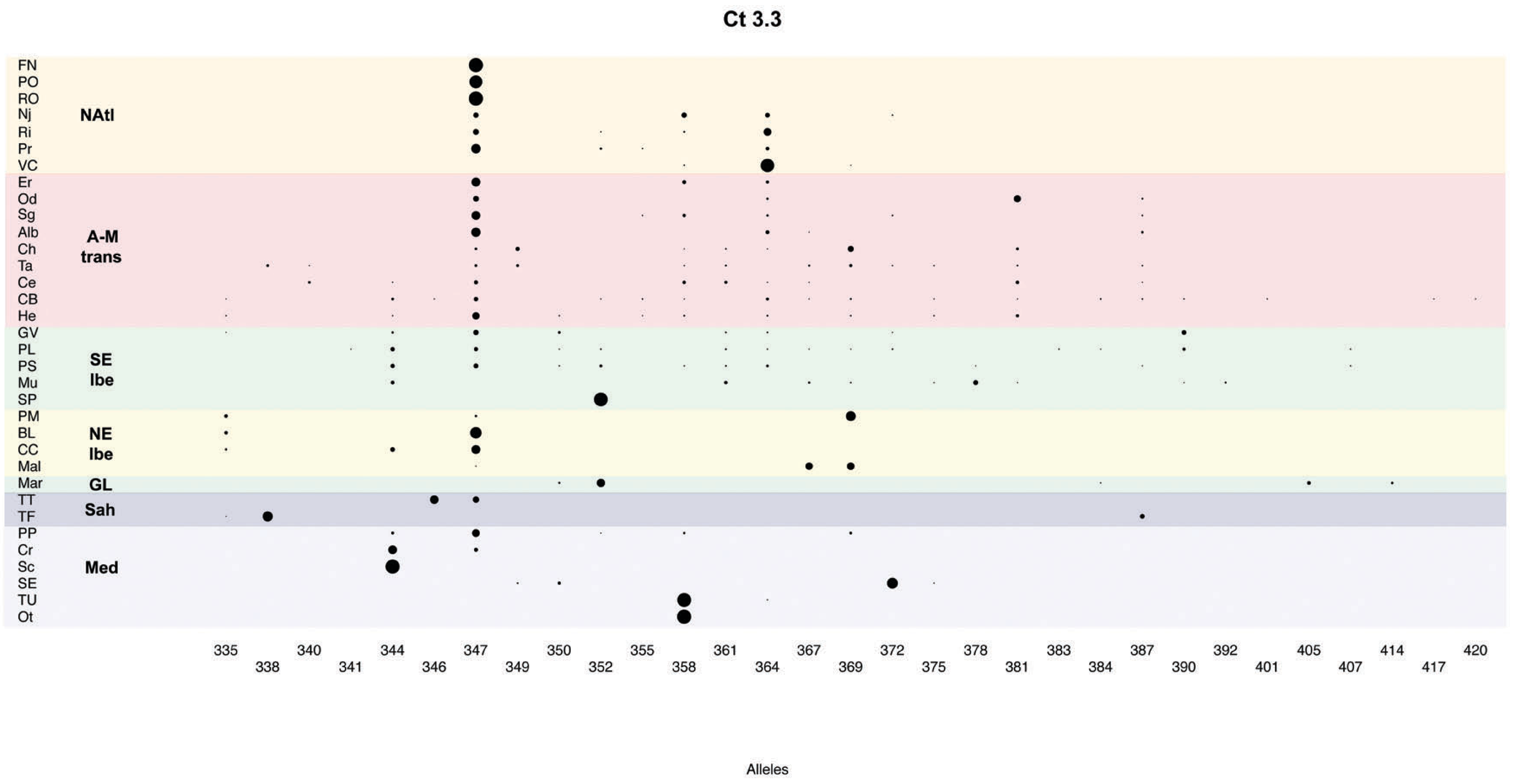
Ct 3.3
FN
PO
RO
Nj
Ri
Pr
VC
NAtl
Er
Od
Sg
Alb
Ch
Ta
Ce
CB
He
A-M
trans
GV
PL
PS
Mu
SP
SE
Ibe
PM
BL
CC
Mal
NE
Ibe
Mar
GL
TT
TF
Sah
PP
Cr
Sc
SE
TU
Ot
Med
335 338 340 341 344 346 347 349 350 352 355 358 361 364 367 369 372 375 378 381 383 384 387 390 392 401 405 407 414 417 420
Alleles

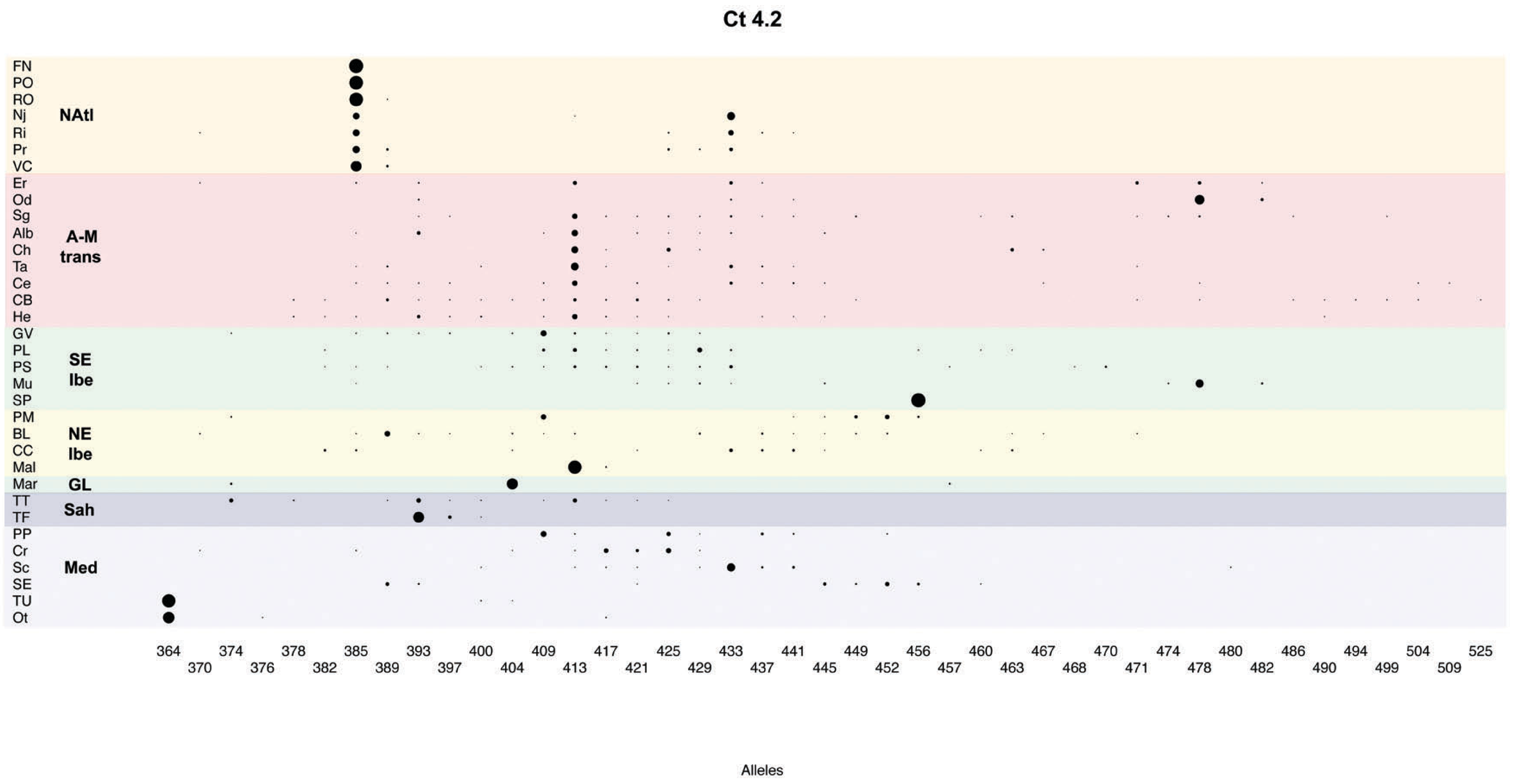

Ct 4.2
FN
PO
RO
Nj
Ri
Pr
VC
NAtl
Er
Od
Sg
Alb
Ch
Ta
Ce
CB
He
A-M trans
GV
PL
PS
Mu
SP
SE Ibe
PM
BL
CC
Mal
NE Ibe
Mar
GL
TT
TF
Sah
PP
Cr
Sc
SE
TU
Ot
Med
364 370 374 376 378 382 385 389 393 397 400 404 409 413 417 421 425 429 433 437 441 445 449 452 456 457 460 463 467 468 470 471 474 478 480 482 486 490 494 499 504 509 525
Alleles

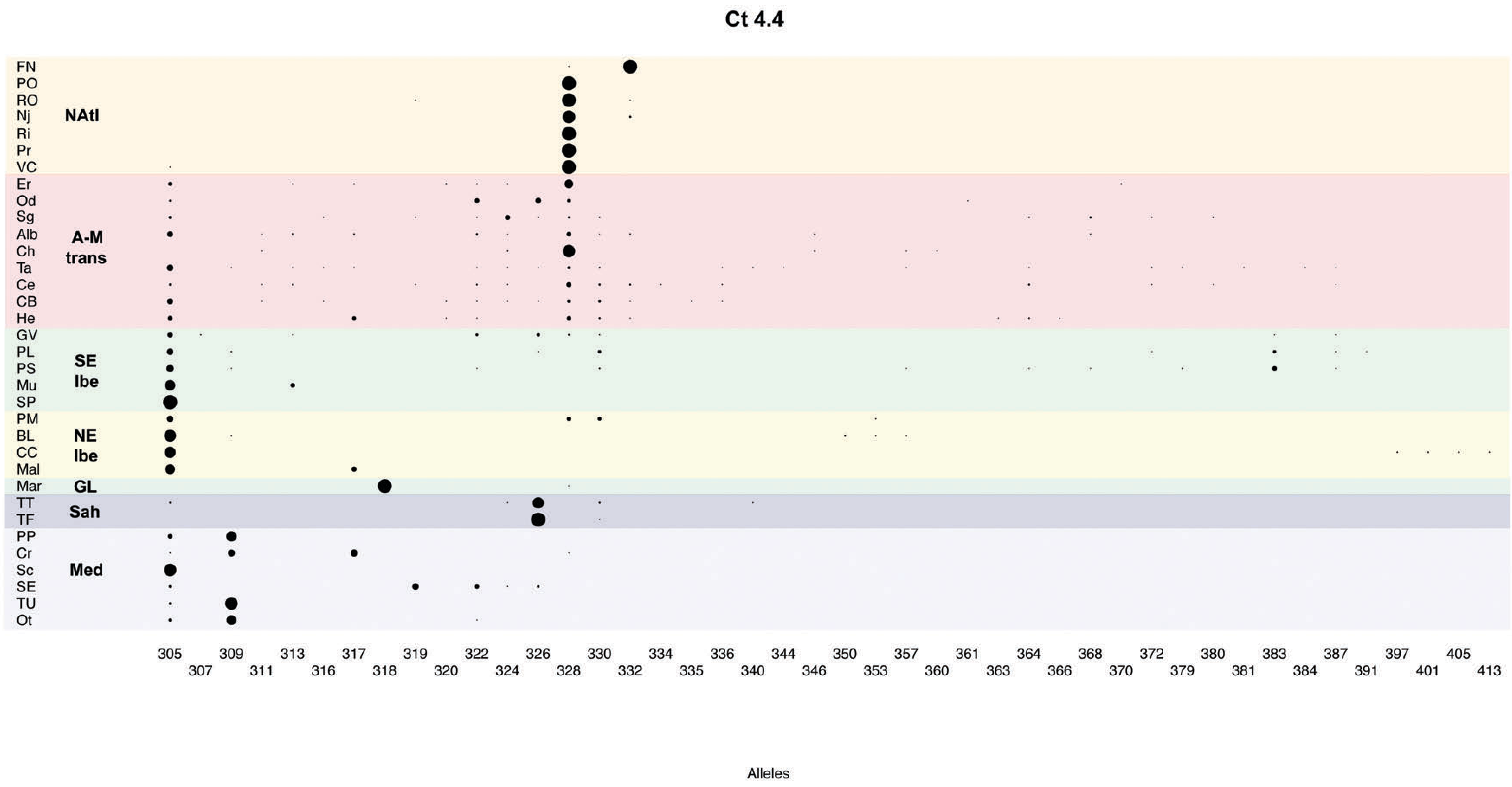

Ct 4.4
FN
PO
RO
Nj
Ri
Pr
VC
NAtl
Er
Od
Sg
Alb
Ch
Ta
Ce
CB
He
A-M trans
GV
PL
PS
Mu
SP
SE Ibe
PM
BL
CC
Mal
NE Ibe
Mar
GL
TT
TF
Sah
PP
Cr
Sc
SE
TU
Ot
Med
305 307 309 311 313 316 317 318 319 320 322 324 326 328 330 332 334 335 336 340 344 346 350 353 357 360 361 363 364 366 368 370 372 379 380 381 383 384 387 391 397 401 405 413
Alleles

Supplementary S4

	D Jost		$F_{ST}/(1-F_{ST})$		
	df	F-values		df	F-values
Distance	1	6.695*	Distance	1	20.650***
Region	2	41.07***	Region	2	41.445***
DistancexRegion	2	0.157	DistancexRegion	2	2.216
Residuals	38		Residuals	38	
Post hoc:			Post hoc:		
Med > A-M trans >Med			Med > Med > A-M trans		

Table S4.- Final analysis of covariance (ANCOVA) model and post hoc results for the effect of shortest sea distance (km) and Region on Jost's D and $F_{ST}/(1-F_{ST})$ genetic distances between populations from the Mediterranean, Atlantic-Mediterranean transition and North Atlantic genetic clusters (see Fig. 2 in the article). To ensure that geographic distance and genetic distances are independent variables, and to avoid any possible bias, just pairwise comparisons in a sea distance range between 150 and 700 km were considered to perform the ANCOVA.

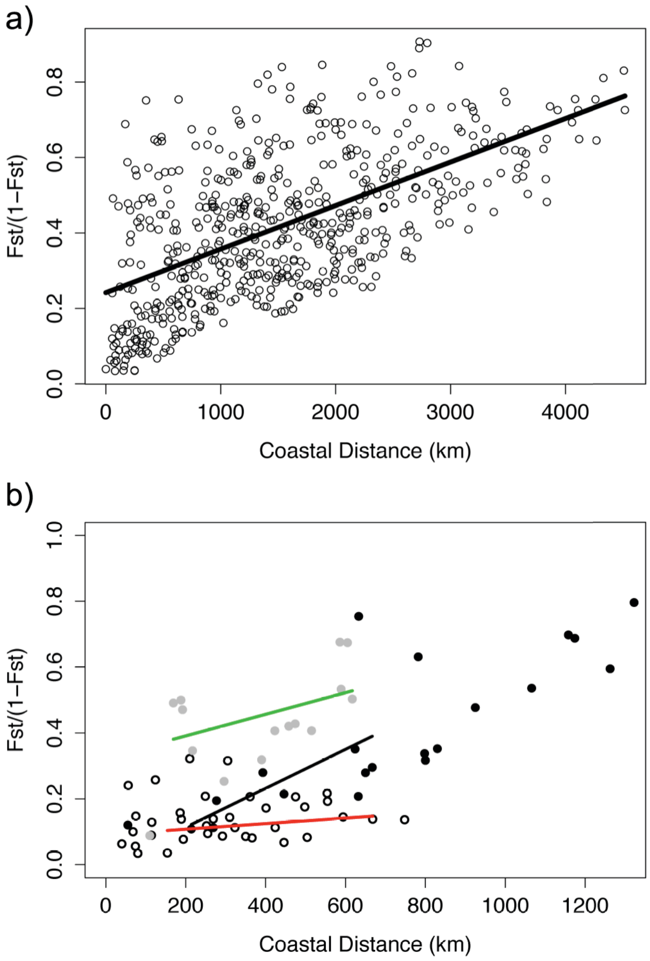

Fig S4.- Relationship between $F_{ST}/(1-F_{ST})$ genetic distance and the shortest sea distance considering: (a) all populations (mantel R = 0.593; p-value<0.001); (b) only North Atlantic (black dots and black line; mantel R = 0.829; p-value <0.001), Atlantic-Mediterranean transition (White dots and red line; mantel R = 0.141; p-value > 0.05) and Mediterranean populations (grey dots and green line; mantel R = 0.596; p-value<0.05). Jost's D results were showed in the article instead $F_{ST}/(1-F_{ST})$, because Jost's D is more appropriate to compare populations with contrasting levels of diversity.
